# Implementing the Use of Collision Cross Section Database for Phycotoxin Screening Analysis

**DOI:** 10.1021/acs.jafc.3c01060

**Published:** 2023-06-22

**Authors:** Maria Mar Aparicio-Muriana, Renato Bruni, Francisco J. Lara, Monsalud del Olmo-Iruela, Maykel Hernandez-Mesa, Ana M. García-Campaña, Chiara Dall’Asta, Laura Righetti

**Affiliations:** † Department of Food and Drug, University of Parma, Parco Area delle Scienze 17/A, Parma 43124, Italy; ‡ Department of Analytic al Chemistry, Faculty of Sciences, University of Granada, Campus Fuentenueva s/n, Granada 18071, Spain; § Laboratory of Organic Chemistry, Wageningen University, Wageningen 6708 WE, the Netherlands; ∥ Wageningen Food Safety Research, Wageningen University & Research, Wageningen 6700 AE, the Netherlands

**Keywords:** ion mobility mass spectrometry, collision cross section, cyanotoxins, phycotoxins, CCS database, BGA dietary supplements

## Abstract

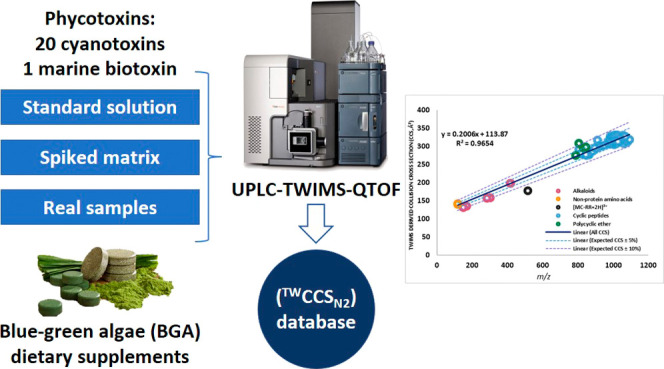

The increased consumption of blue-green algae (BGA)-based dietary supplements has raised concern about their food safety, especially about cyanotoxin presence. The hyphenation of liquid chromatography with ion mobility mass spectrometry represents a relevant tool to screen several compounds in a large variety of food matrices. In this work, ultrahigh-performance liquid chromatography coupled to traveling wave ion mobility spectrometry/quadrupole time-of-flight mass spectrometry (UHPLC-TWIMS-QTOF) was employed to establish the first comprehensive TWIMS-derived collision cross section database (^TW^CCS_N2_) for phycotoxins. The database included 20 cyanotoxins and 1 marine toxin. Accurate *m*/*z*, retention times, and ^TW^CCS_N2_ values were obtained for 81 adducts in positive and negative electrospray (ESI^+^/ESI^–^) modes. Reproducibility and robustness of the ^TW^CCS_N2_ measurements were determined to be independent of the matrix. A screening was carried out on 19 commercial BGA dietary supplements of different composition. Cyanotoxins were confidently identified in five samples based on retention time, *m*/*z*, and ^TW^CCS_N2_.

## 1 Introduction

**Figure 1:**
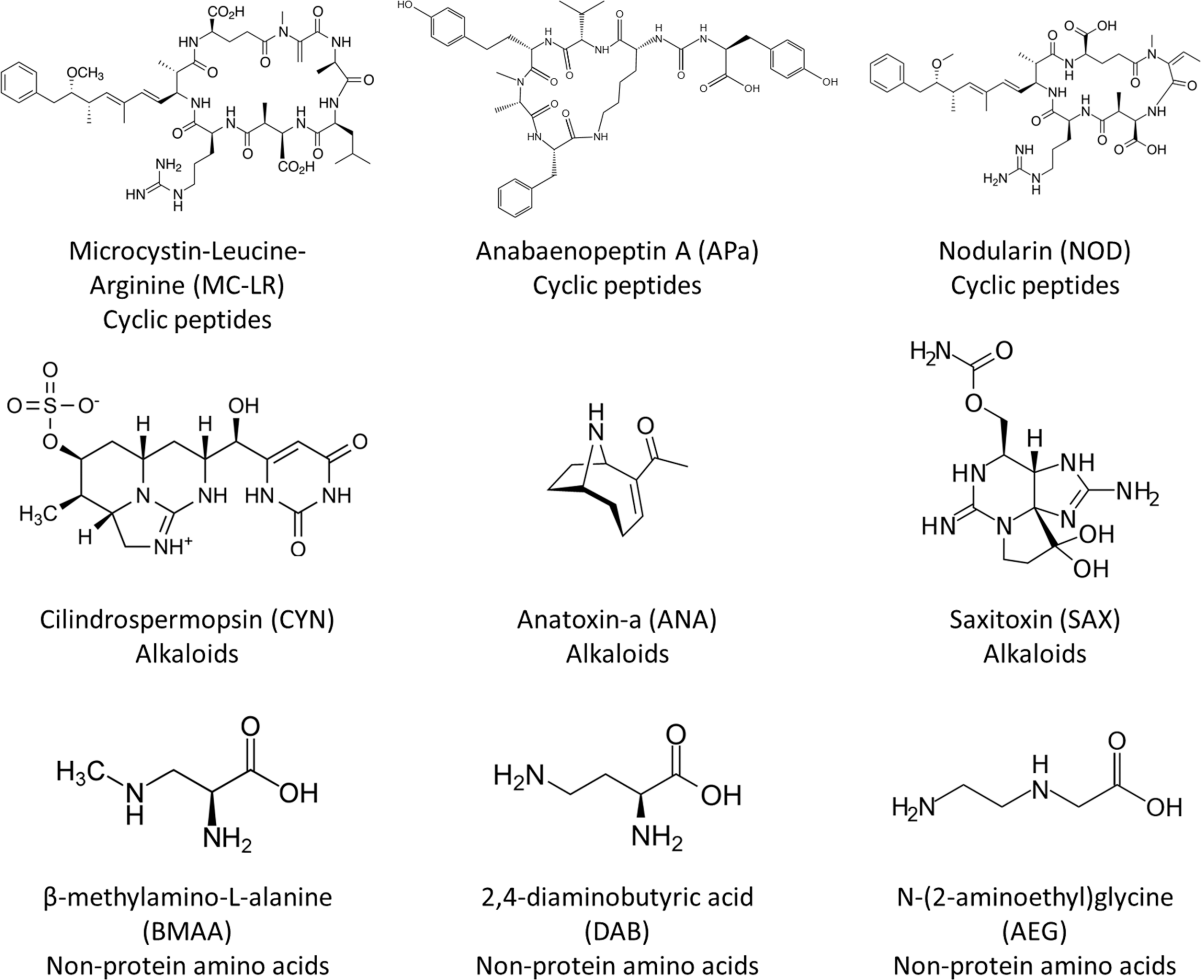
Structures of several cyanotoxins from cyclic peptide, alkaloid, and nonprotein amino acid families.

The dietary consumption of blue-green algae (BGA) has increased as a consequence of their purported nutritional benefits and their use in dietary supplements.^1^ BGA, also known as cyanobacteria, constitute a diverse, polyphyletic group of oxygenic photosynthetic prokaryotes most commonly found in freshwater, which are employed in different applications in industry, including the production of food, feed, biofertilizers, and cosmetics.^2^ Their cultivation is performed both in bioreactors and open-air reservoirs, and their unique nature may influence the quality and safety profile of final products. However, cyanobacteria may also release toxins into the environment. Under certain temperature, light, salinity and pH conditions, and high nutrient availability, cyanobacteria can produce massive biomass growth (“blooms”), which causes numerous problems, and it is of particular concern when the cyanobacteria strains are toxin-producing.^3^ These situations are specially favored by global warming caused by climate change,^4^ and by increased water eutrophication caused by current human activity,^5^ which emphasize the need of a comprehensive monitoring of the occurrence of such substances. At the same time, other organisms may simultaneously grow and contaminate the desired BGA strains, leading to the potential co-occurrence of unwanted, toxic species such as dinoflagellates, able to release marine biotoxins,^6^ and whose visual recognition is made difficult by the microscopic nature of cyanobacteria and by the presence of very similar species with different harmful potential.^7^ Cyanotoxins are toxic secondary metabolites generated by some species of cyanobacteria that pose an emerging threat as they can bioaccumulate in the aquatic organisms and be transferred throughout the food chain.^8^ Cyanotoxins include a large variability of chemical scaffolds, ranging from alkaloids to nonprotein amino acids, from cyclic peptides to polycyclic ethers.^9^Figure 1 shows the structure differences of some cyanotoxins from cyclic peptide, alkaloid, and nonprotein amino acid families. The structure of all toxins can be observed in Supporting Material file, Table S1. Some of them can be produced by different types of cyanobacteria genera as well as one cyanobacteria genera can produce different types of cyanotoxins.^10^ While the use of BGA is increasing in feeds with an indirect entry in human food chains, the most direct exposure comes from dietary supplements, in which a variety of cyanotoxins have been found recently.^11−18^ As mentioned before, other toxin-producing eukaryote algae, such as dinoflagellates, cohabit with cyanobacteria in aquatic environments; thus, a variety of phycotoxins can be found together forcing on multiple approaches to obtain a reliable overview. Monitoring the safety of BGA-derived foods is made further relevant by the increasing rate of novel food applications regarding these organisms.^7^

Most commercial BGA dietary supplements come from *Arthrospira platensis* Gomont (commonly known as Spirulina) and *Aphanizomenon flos-aquae* (Linnaeus) Ralfs ex Bornet and Flahault filamentous species.^18^ The World Health Organization (WHO) established a recommended tolerable daily intake (TDI) of 0.04 μg/kg body weight per day and a provisional guideline value of 1 μg/L only for the hexapeptide microcystin leucine arginine (MC-LR) in drinking water.^19^ However, to date, there are no official guidelines that regulate the presence of cyanotoxins in dietary supplements, even if MC-LR has been classified as possibly carcinogenic to humans (group 2B) by the International Agency for Research on Cancer (IARC).^20^ Only the Oregon Health Division and the Oregon Department of Agriculture set a regulatory limit of 1 μg/g for microcystins in BGA-containing products.^21^ Contaminated dietary supplements can be a major exposure source to microcystins (MCs). However, it is hard to estimate the actual contribution of these products in an overall health risk context due to different levels of MCs contamination and lack of information on the extent of use of supplements. Therefore, the acquisition of new occurrence data is relevant to collect more information on the presence of MCs and other phycotoxins in dietary supplements.

Within this situation, analytical issues are represented by the pleiotropic nature of contaminants and by the limited knowledge of potentially harmful species. The use of ultrahigh-performance liquid chromatography (UHPLC) coupled to low-resolution mass spectrometry detection systems (LRMS) has been therefore one of the most employed techniques to determine cyanotoxins in most food matrices.^22,23^ However, LRMS shows some limitations due to the acquisition mode, such as the time-consuming compound-depending optimization of the acquisition parameters,^24^ or the incapacity to conduct retrospective data analysis.^25^ The use of high-resolution mass spectrometry (HRMS) provides a powerful alternative as it allows the settlement of these handicaps. In fact, the main improvement of HRMS-based approaches is the acquisition of high-resolution full-scan mass spectrometry (MS) data, which aids a retrospective data analysis of nontarget compounds without reinjecting the samples. In addition, HRMS allows the combination of target and nontarget analyses, providing significant improvements in screening and profiling of complex mixtures.^26^ In this line, the hyphenation of ion mobility spectrometry (IMS) with HRMS and its introduction in traditional UHPLC–MS workflows have emerged as a powerful technique that enhances the quality, quantity, and specificity of the information.^27^ IMS instrumentation allows the measurement of the collision cross section (CCS, Ω), which is considered a molecular descriptor as it is an intrinsic property of each molecule, whose value is directly linked to the chemical structure and three-dimensional conformation, and it is not affected by the sample matrix. Therefore, CCS can be used as an additional separation dimension alongside with the traditional parameters such as retention time and accurate *m*/*z* to improve screening capacity, selectivity, and sensitivity, as already demonstrated for different classes of food contaminants.^28−31^ In addition, the use of CCS can provide an extra level of confidence in the identification reducing the number of false positives, as different isomers or conformers of the same molecule can have different CCS values. This can be particularly useful for the identification of structural isomers, which can be difficult to distinguish based on retention time and accurate mass measurements alone and when dealing with complex mixtures or unknown compounds. Moreover, in suspect screening and nontargeted analysis, when the retention time of the compounds is not available, the use of CCS values can be a useful parameter to improve the confidence of tentative identification. In this sense, the incorporation of CCS as an identification parameter requires the use of reliable CCS databases that provide these experimental values for as many molecules as possible. However, several toxic compounds and residues remain uncharacterized in terms of CCS; thus, further efforts are needed to overcome the lack of appropriate databases.

With this background, the main goal of the present work was to generate the first TWIMS-derived CCS database for phycotoxins, starting from 21 compounds of different chemical natures (20 cyanotoxins and 1 marine biotoxin), aiming to implement the use of IMS in phycotoxin screening workflows and to extend the current IMS knowledge about natural toxins. To establish the potential of the CCS as a molecular descriptor, CCS values were measured through different experimental conditions. Likewise, the strengths and challenges that arose from the developed library were discussed. Finally, as a proof of concept, the database was applied to the qualitative screening of BGA dietary supplements of different composition.

## 2 Materials and Methods

### 2.1 Chemicals

HPLC-grade methanol (MeOH) was purchased from Sigma-Aldrich (Taufkirchen, Germany), bidistilled water was obtained using a Milli-Q System (Millipore, Bedford, MA, USA), MS-grade formic acid was purchased from Fisher Chemical (Thermo Fisher Scientific Inc., San Jose, CA, USA), and ammonium formiate was obtained from Sigma-Aldrich (St. Luis, MO, USA). Leucine-enkephalin [186006013] used as lock mass solution and Major Mix IMS/TOF Calibration Kit [186008113] for mass and CCS calibration were purchased from Waters (Manchester, UK).

Analytical standards of phycotoxins were purchased individually from Enzo Life Sciences, Inc. (Lausen, Switzerland), Sigma-Aldrich (Darmstadt, Germany), and Cayman Chemicals (Michigan, USA). Detailed information such as common names, toxicity, structure, etc. of the targeted toxins are included in Table S1. Stock standard solutions of 50 or 25 μg·mL^–1^ were prepared by adding 1 mL of desired solvent directly into the vial of toxin supplied by the manufacturer and gently swirling the vial to dissolve the toxin. Cylindrospermopsin (CYN), microcystin-leucine-arginine (MC-LR), microcystin-tyrosine-arginine (MC-YR), microcystin-tryptophan-arginine (MC-WR), microcystin-leucine-alanine (MC-LA), microcystin-leucine-tyrosine (MC-LY), microcystin-leucine-tryptophan (MC-LW), microcystin-leucine-phenilalanine (MC-LF), microcystin-homoisoleucine-arginine (MC-HliR), microcystin-homotyrosine-arginine (MC-HtyR), [DAsp3]-microcystin-leucine-arginine ([DAsp3]-MC-LR), anabaenopeptin A (APa), and anabaenopeptin B (APb) were prepared in 100% MeOH; nodularin (NOD) was prepared in H_2_O:MeOH (1:1); microcystin-arginine-arginine (MC-RR) was prepared in H_2_O:MeOH (20:80); okadaic acid (OA) was prepared in 100% ethanol, anatoxin-a (ANA), and the isomers β-methylamino-lalanine (BMAA); 2,4-diaminobutyric acid (DAB) and *N*-(2-aminoethyl)glycine (AEG) were prepared in H_2_O; and saxitoxin (SAX) was prepared in 0.003 M HCl. Stock solutions were stored in the dark at −20 °C. Intermediate standard solutions of each compound at 2.5 μg·mL^–1^ were prepared by dilution of the stock solutions with the corresponding solvent for each toxin.

### 2.2 BGA-Based Dietary Supplement Samples

BGA-derived dietary supplements were obtained from several brands and different sources, largely on internet but also from local retail stores in Granada (Spain) and Parma (Italy). They were sold as tablets, capsules, powder, and liquid form, thus presenting different matrices, as they were composed of different cyanobacteria species in different ratios. In addition, some of them were also formulated with separate excipients along with other components. Detailed information on samples with their forms, composition, and daily doses are listed in Table S2. All the samples were analyzed before their expiration date.

### 2.3 Sample Preparation

Tablets were pulverized with a mortar and pestle to make a fine powder. For capsules, five samples were opened and the contents mixed and triturated in a mortar and pestle. Aliquots of powdered sample were submitted to the protocol previously employed by van Pamel et al.^32^ for the extraction of plant toxins and cyanotoxins in dietary supplements. Briefly, 0.5 g of each sample was weighted and introduced in a conical polypropylene centrifuge tube and 5 mL of 75% MeOH in water was added (sample:extraction solvent, 1:10). After vortex shaking for 1 min, samples were mechanically shaken for 10 min, placed in an ultrasonic bath for 15 min at room temperature, and mechanically shaken again for 10 min. After that, the extracts were centrifuged for 10 min at 9000 rpm at room temperature, and finally, 100 μL of supernatant was diluted up to 375 μL of H_2_O with 0.1% formic acid to obtain a ratio 80:20 H_2_O:MeOH. The diluted extract was transferred to a vial and injected into the UHPLC-TWIMS-QTOF system.

### 2.4 UHPLC Analysis Conditions

An ACQUITY I-Class UHPLC separation system was employed. For the chromatographic separation, two different columns and methods were employed depending on the nature of the toxins. On the one hand, an Acquity UHPLC BEH C18 column (2.1 mm × 100 mm, 1.7 μm particle size) (Waters, Manchester, U.K.) was employed for all the MC congeners, NOD, APa, APb, CYN, SAX, and OA. The chromatographic method, based on a published application note from Waters,^33^ employed water with 0.1% of formic acid as solvent A and MeCN with 0.1% of formic acid as solvent B. The separation was achieved using the following gradient mode: 0 min, 0% B flow 0.4 mL/min; 1.5 min, 0% B flow 0.4 mL/min; 6.5 min, 80% B flow 0.4 mL/min; 6.6 min, 100% B flow 0.5 mL/min; 11 min, 100% B flow 0.5 mL/min; 11.1 min, 0% B flow 0.4 mL/min; and 14 min, 0% B flow 0.4 mL/min. The column and autosampler were maintained at 45 and 10 °C, respectively, and 2 μL of extract was injected. On the other hand, an Atlantis Premier BEH Z-HILIC column (2.1 mm × 100 mm, 1.7 μm particle size) from Waters (Manchester, U.K.) was used for ANA and for the nonprotein amino acid isomers BMAA, DAB, and AEG. The mobile phase consisted of 10 mM ammonium formiate with 0.3% formic acid in water (A) and 0.3% formic acid in MeCN (B). The separation was performed using the following gradient mode: 0 min, 95% B; 2 min, 95% B; 10 min, 50% B; 11 min, 50% B; 12 min, 95% B; and 15 min, 95% B at a flow rate of 0.4 mL/min. The column and autosampler were maintained at 45 and 10 °C, respectively, and 4 μL of extract was injected. Figure S1A,B shows the chromatograms of the analytes with the retention times using both reversed phase and HILIC methods, respectively.

An ACQUITY I-Class UHPLC separation system was employed. For the chromatographic separation, two different columns and methods were employed depending on the nature of the toxins. On the one hand, an Acquity UHPLC BEH C18 column (2.1 mm × 100 mm, 1.7 μm particle size) (Waters, Manchester, U.K.) was employed for all the MC congeners, NOD, APa, APb, CYN, SAX, and OA. The chromatographic method, based on a published application note from Waters,^33^ employed water with 0.1% of formic acid as solvent A and MeCN with 0.1% of formic acid as solvent B. The separation was achieved using the following gradient mode: 0 min, 0% B flow 0.4 mL/min; 1.5 min, 0% B flow 0.4 mL/min; 6.5 min, 80% B flow 0.4 mL/min; 6.6 min, 100% B flow 0.5 mL/min; 11 min, 100% B flow 0.5 mL/min; 11.1 min, 0% B flow 0.4 mL/min; and 14 min, 0% B flow 0.4 mL/min. The column and autosampler were maintained at 45 and 10 °C, respectively, and 2 μL of extract was injected. On the other hand, an Atlantis Premier BEH Z-HILIC column (2.1 mm × 100 mm, 1.7 μm particle size) from Waters (Manchester, U.K.) was used for ANA and for the nonprotein amino acid isomers BMAA, DAB, and AEG. The mobile phase consisted of 10 mM ammonium formiate with 0.3% formic acid in water (A) and 0.3% formic acid in MeCN (B). The separation was performed using the following gradient mode: 0 min, 95% B; 2 min, 95% B; 10 min, 50% B; 11 min, 50% B; 12 min, 95% B; and 15 min, 95% B at a flow rate of 0.4 mL/min. The column and autosampler were maintained at 45 and 10 °C, respectively, and 4 μL of extract was injected. Figure S1A,B shows the chromatograms of the analytes with the retention times using both reversed phase and HILIC methods, respectively.

### 2.5 TWIMS-QTOF Conditions

The ACQUITY I-Class UHPLC separation system was coupled to a Vion IMS-QTOF mass spectrometer (Waters, Manchester, UK) equipped with an ESI interface. The IMS-MS system consists of a hybrid quadrupole orthogonal acceleration time-of-flight mass spectrometer, where the mobility cell, which is a stacked ring ion guide, is placed before the quadrupole mass filter. The mass spectrometry detection was conducted in both positive and negative electrospray ionization mode in the mass range of *m*/*z* 50–1100 with a scan time of 0.15 and 0.30 s for the reversed phase and HILIC method, respectively. Argon was used as the collision gas, and nitrogen was used as the ion mobility gas. The IMS gas flow rate was 90 mL/min (3.2 mbar), a wave velocity of 650 *m*/*s*, and a wave height of 40 V.

For the reversed phase method, parameters related to source conditions were set as follows: capillary voltage, 2.5 kV; cone voltage, 40 V; source temperature, 150 °C; desolvation temperature, 600 °C; desolvation gas flow, 950 L/h; and cone gas flow, 50 L/min. In data-independent acquisition mode, using IM technology (designed as high-definition MS^E^ (HDMS^E^) in the case of our particular instrumentation), two data channels are acquired simultaneously in a single run. The fragmentation of precursor ions (monitored from 50 to 1100 *m*/*z*) is minimized in the low-energy channel, so it is used to monitor the protonated and deprotonated molecules and other formed adducts. A collision energy ramp is applied in the high-energy channel to induce fragmentation of precursor ions traveling through the collision cell. The low-energy spectra were acquired at CE of 6 V for both ESI+ and ESI–, while high-energy spectra were acquired with a ramp of the transfer CE from 30 to 80 V.

For the HILIC method, parameters related to source conditions were set as follows: capillary voltage, 1 kV; cone voltage, 30 V; source temperature, 150 °C; desolvation temperature, 450 °C; desolvation gas flow, 800 L/h; and cone gas flow, 50 L/min. The low-energy spectra were acquired at a CE of 6 eV for both ESI+ and ESI–, while high-energy spectra were acquired with a CE ramp from 10 to 50 eV.

Lock mass correction was performed by infusing a solution of leucine-encephalin [M + H]^+^ (*m*/*z* 556.2766, calibration kit from Waters) at a concentration of 200 pg/μL (infusion rate, 10 μL/min) and acquired every 2.5 min to provide a real-time single-point mass and CCS calibration.

### 2.6 Creation of the CCS Database

Phycotoxin standard mixes were prepared at four different concentration levels (10, 50, 100, and 500 μg/L), and 2 and 4 μL were injected in the reversed phase and HILIC method, respectively. ^TW^CCS_N2_ values were obtained from the average of nine replicates for 500 and 100 μg/L standard mixtures plus three replicates for 50 and 10 μg/L standard mixtures, employing a Vion IMS quadrupole time-of-flight (QTOF) instrument (resolution ∼20 Ω/ΔΩ fwhm). ^TW^CCS_N2_ values were measured using nitrogen as drift gas and were experimentally determined by the application of CCS calibration curves created using the Major Mix CCS calibration solution for both ESI+ and ESI– mode. TWIMS calibration procedure has been previously described,^34^ and it is automatically performed by UNIFI 1.8 software (Waters; Manchester, UK). The Major Mix calibration solution contained poly-dl-alanine, Ultramark 1621, low-molecular-weight acids, and other small molecules. The calibrants covered a *m*/*z* range from 152.0706 to 1921.9459 Da, and CCS range from 130.4 to 372.6 Å^2^ in positive mode and a mass range from 150.0561 to 1965.9369 Da, and a CCS range from 131.5 to 367.2 Å^2^ in negative mode. Major Mix was prepared in 50:50 (v:v) water:acetonitrile with 0.1% formic acid. The exact composition of the different calibration solutions is reported in Tables S3 and S4. CCS calibration was carried out considering singly charged ions, so TWIMS-derived CCS values were only applicable to singly charged ions. All the ionized species detected for each toxin were identified with a deviation lower than 5 ppm in relation to their exact mass.

### 2.7 Software and Data Analysis

Data acquisition was conducted using UNIFI 1.8 software (Waters; Manchester, UK), which also provides the ^TW^CCS_N2_ values. Theoretical CCS values were also predicted by three different machine learning tools named AllCCS (http://allccs.zhulab.cn/),^35^ CCSbase (https://ccsbase.net/),^36^ and MetCCS Predictor (http://www.metabolomics-shanghai.org/MetCCS/).^37^ The molecular descriptors required for CCS prediction were obtained from the human metabolome database (HMDB, http://www.hmdb.ca/)^38^ and PubChem database (https://pubchem.ncbi.nlm.nih.gov/).^39^

## 3 Results and Discussion

To implement IMS in routine MS-based phycotoxin workflows, searchable databases with CCS values and accurate mass values need to be generated. This work reports the first ^TW^CCS_N2_ database for cyanotoxins, which encompasses compounds from different families, including cyclic peptides (*n* = 14), alkaloids (*n* = 3), and nonprotein amino acids (*n* = 3). In addition to cyanotoxins, OA, which is the main representative of the marine biotoxins,^40^ was also characterized because they often coexist in marine environments. Overall, 21 phycotoxins were characterized in terms of ^TW^CCS_N2_. All ^TW^CCS_N2_ values were collected from commercially available standards (Table S1). Various parameters and instrumental conditions were tested to validate the database, and ^TW^CCS_N2_ values were also measured in spiked dietary supplement extracts to prove the reliability of the CCS measurements. Moreover, its applicability to the phycotoxin screening analysis of BGA-derived dietary supplements was investigated.

### 3.1 Phycotoxin ^TW^CCS_N2_ Database

All phycotoxins (*n* = 21) were characterized in both positive and negative ionization modes. ^TW^CCS_N2_ measurements were carried out through several replicates (nine times the standard solutions of 500 and 100 μg/L plus three times the standard solutions of 50 and 10 μg/L). The developed database provides the ^TW^CCS_N2_ of the most abundant ion observed for each toxin, but it also offers information about all the identified adducts observed for each compound in both positive and negative ionization modes (e.g., [M+H]^+^, [M+Na]^+^, [M+K]^+^, [M+H–H_2_O]^+^, [M–H]^−^, and [M–H–H_2_O]^−^) as well as their influence in the drift time. The CCS of the most intense adduct was observed at all injected concentration levels; however, the ^TW^CCS_N2_ of all other adducts could not be determined for the 50 and 10 μg/L standard solutions due to their low peak intensity. Overall, a total of 81 ions (considering protonated and deprotonated molecules, cationic and anionic adducts) have been identified and characterized in terms of *m*/*z* and CCS. In detail, protonated adducts were detected for all compounds except for the marine biotoxin okadaic acid and three amino acid isomer cyanotoxins. Complete information of the investigated toxins, the observed ions under positive and negative ESI conditions, their *m*/*z* and ^TW^CCS_N2_ values can be found in Table 1. In all cases, high reproducibility was observed with relative standard deviations (RSDs) lower than 0.3% for 71% of the compounds, being 0.72 and 0.55%, the highest values obtained for positive and negative ionization mode, respectively.

**Table 1:** CCS Database for Phycotoxins Using N_2_ as Drift Gas (*n* = 24)

compound	adduct	theoretical exact *m*/*z*	experimental ^TW^CCS_N2_ (Å^2^)	SD	RSD (%)
β-methylamine-l-alanine	[M–H]^−^	117.0670	140.6	0.56	0.40
2,4-diaminobutyric acid	[M–H]^−^	117.0670	140.7	0.78	0.55
*N*-(2-aminoethyl)glycine	[M–H]^−^	117.0670	141.9	0.55	0.39
anatoxin-a	[M+H]^+^	166.1227	136.1	0.32	0.24
anatoxin-a	[M+H–H_2_O]^+^	148.1121	132.6	0.19	0.14
saxitoxin	[M+H]^+^	300.1415	159.6	0.31	0.19
saxitoxin	[M+H–H_2_O]^+^	282.1309	157.2	0.29	0.18
cylindrospermopsin	[M+H]^+^	416.1235	198.6	0.42	0.21
cylindrospermopsin	[M–H]^−^	414.1089	198.9	0.28	0.14
okadaic acid	[M–H]^−^	803.4582	308.4	0.36	0.12
okadaic acid	[M+Na]^+^	827.4558	296.9	0.26	0.09
okadaic acid	[M+K]^+^	843.4297	298.9	0.36	0.12
okadaic acid	[M+H–H_2_O]^+^	785.4476	275.5	0.40	0.14
nodularin	[M+H]^+^	825.4505	296.5	0.68	0.23
nodularin	[M–H]^−^	823.4359	288.8	0.54	0.19
nodularin	[M+Na]^+^	847.4325	274.9	0.99	0.36
nodularin	[M+K]^+^	863.4070	277.3	0.90	0.32
nodularin	[M–H–H_2_O]^−^	805.4249	291.9	1.43	0.49
anabaenopeptin B	[M+H]^+^	837.4618	278.3	0.45	0.16
anabaenopeptin B	[M–H]^−^	835.4472	286.4	0.86	0.30
anabaenopeptin B	[M+Na]^+^	859.4438	282.2	1.14	0.41
anabaenopeptin B	[M–H–H_2_O]^−^	817.4361	280.5	0.99	0.35
anabaenopeptin A	[M+H]^+^	844.4240	279.2	0.53	0.19
anabaenopeptin A	[M–H]^−^	842.4094	278.3	0.44	0.16
anabaenopeptin A	[M+Na]^+^	866.4060	285.5	0.55	0.19
anabaenopeptin A	[M+K]^+^	882.3804	286.7	1.01	0.35
anabaenopeptin A	[M+H–H_2_O]^+^	826.4134	277.8	1.10	0.39
microcystin-LA	[M+H]^+^	910.4921	296.0	0.79	0.27
microcystin-LA	[M–H]^−^	908.4775	317.2	0.49	0.15
microcystin-LA	[M+Na]^+^	932.4741	301.3	0.74	0.25
microcystin-LA	[M+K]^+^	948.4485	303.2	0.87	0.29
microcystin-LA	[M+H–H_2_O]^+^	892.4815	296.0	1.81	0.61
[D-Asp3]-microcystin-LR	[M+H]^+^	981.5409	305.9	1.17	0.38
[D-Asp3]-microcystin-LR	[M–H]^−^	979.5253	324.8	0.41	0.13
[D-Asp3]-microcystin-LR	[M+Na]^+^	1003.5229	304.7	1.27	0.42
microcystin-LF	[M+H]^+^	986.5234	309.2	0.80	0.26
microcystin-LF	[M–H]^−^	984.5088	329.7	0.51	0.15
microcystin-LF	[M+Na]^+^	1008.5054	316.3	0.63	0.20
microcystin-LF	[M+K]^+^	1024.4798	319.2	1.03	0.32
microcystin-LF	[M+H–H_2_O]^+^	968.5128	310.2	1.41	0.45
microcystin-LR	[M+H]^+^	995.5561	309.3	0.60	0.19
microcystin-LR	[M–H]^−^	993.5415	326.9	0.50	0.15
microcystin-LR	[M+Na]^+^	1017.5381	307.3	0.68	0.22
microcystin-LR	[M+K]^+^	1033.5125	318.9	1.02	0.32
microcystin-LR	[M+H–H_2_O]^+^	977.5455	310.2	1.41	0.45
microcystin-LY	[M+H]^+^	1002.5183	313.5	0.71	0.23
microcystin-LY	[M–H]^−^	1000.5037	326.1	0.53	0.16
microcystin-LY	[M+Na]^+^	1024.5003	320.0	0.61	0.19
microcystin-LY	[M+H–H_2_O]^+^	984.5077	313.8	1.33	0.43
microcystin-HilR	[M+H]^+^	1009.5722	314.7	0.86	0.27
microcystin-HilR	[M–H]^−^	1007.5566	331.5	0.40	0.12
microcystin-HilR	[M+Na]^+^	1031.5542	312.1	0.65	0.21
microcystin-LW	[M+H]^+^	1025.5343	317.3	0.69	0.22
microcystin-LW	[M–H]^−^	1023.5197	332.1	0.38	0.11
microcystin-LW	[M+Na]^+^	1047.5163	320.7	0.49	0.15
microcystin-LW	[M+K]^+^	1063.4907	322.0	0.96	0.30
microcystin-LW	[M+H–H_2_O]^+^	1007.5237	317.2	1.03	0.32
microcystin-RR	[M+2H]^2+^	514.7550	177.5	0.23	0.13
microcystin-RR	[M+H]^+^	1038.5731	316.4	0.62	0.20
microcystin-RR	[M–H]^−^	1036.5585	327.4	0.69	0.21
microcystin-RR	[M+Na]^+^	1060.5551	307.4	0.75	0.24
microcystin-YR	[M+H]^+^	1045.5353	318.0	0.63	0.20
microcystin-YR	[M–H]^−^	1043.5207	322.7	0.44	0.14
microcystin-YR	[M+Na]^+^	1067.5173	316.6	0.75	0.24
microcystin-YR	[M–H–H_2_O]^−^	1025.5096	313.6	0.58	0.18
microcystin-HtyR	[M+H]^+^	1059.5510	316.4	0.69	0.22
microcystin-HtyR	[M–H]^−^	1057.5364	331.2	0.48	0.14
microcystin-HtyR	[M+Na]^+^	1081.5330	312.0	0.86	0.28
microcystin-HtyR	[M+K]^+^	1092.5244	319.7	2.30	0.72
microcystin-WR	[M+H]^+^	1068.5513	320.0	0.77	0.24
microcystin-WR	[M–H]^−^	1066.5367	328.2	0.46	0.14
microcystin-WR	[M+Na]^+^	1090.5333	319.5	0.92	0.29

Being the present one the first database developed for phycotoxins, the comparison of CCS measurements presented here with other previously reported using TWIMS, drift tube ion mobility spectrometry (DTIMS), or trapped ion mobility spectrometry (TIMS) technology is not possible to this day. The only cyanotoxin that had already been characterized in terms of CCS is SAX. The CCS value of the protonated SAX adduct obtained in the present work varied by less than 1.1% from the value previously reported using the TWIMS cell of the Synapt G2 HDMS instrument and N_2_ as buffer gas,^41^ even though the calibration mix employed previously was polyalanine instead of Major Mix. Another study that took advantage of IMS to enhance cyanotoxin determination employed differential mobility spectrometry (DMS) as an ion filter after HILIC separation and ESI and before MS/MS detection for the separation of BMAA and its isomers.^42^ However, no CCS values are determined with this IMS instrumentation.

**Figure 2:**
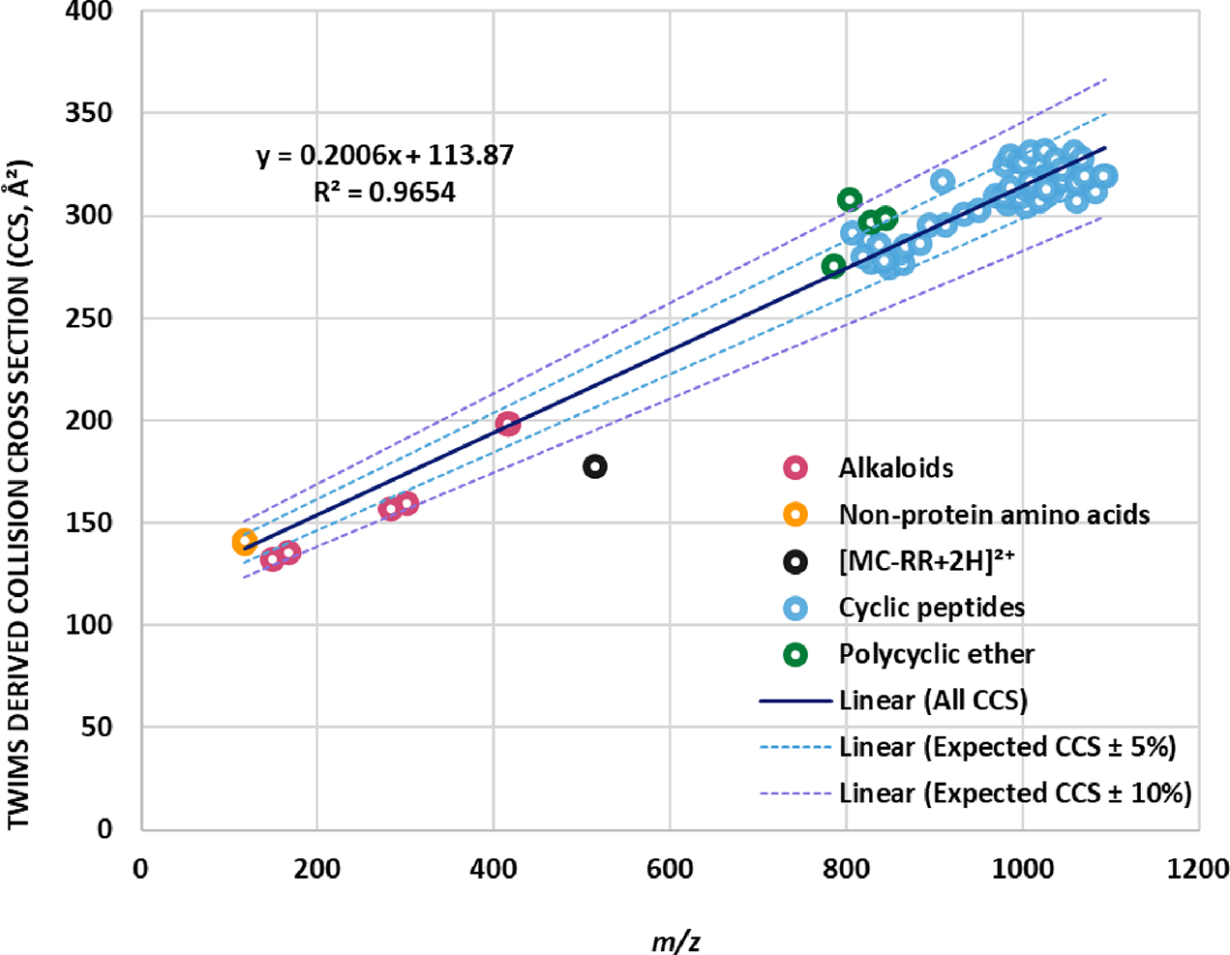
Correlations of *m*/*z* and measured TWIMS-derived CCS values of 21 phycotoxins determined in ESI+ and ESI– modes.

As the CCS is a molecular characteristic closely related to the *m*/*z* ratio, correlation between both parameters is often expected for compounds belonging to the same chemical family or with similar structures.^43^ To analyze the correlation between CCS values and *m*/*z*, the experimentally determined CCSs of all singly charged adducts detected were plotted as a function of *m*/*z* (Figure 2). The range of *m*/*z* and CCS values obtained showed the structural diversity among the target toxins, as compounds that share structural characteristics showed a trend in their ion mobilities. Thus, two main groups of data were observed depending on their toxin family. The first one had lower values of CCS and *m*/*z* (below 198.6 Å^2^ and 416.1, respectively), which corresponds with the alkaloid group of toxins (anatoxin, cylindrospermopsin, and saxitoxin) (Figure S2A). The second group of data, encompassing the cyclic peptides toxins (microcystins, nodularin, and anabaenopeptins), presented higher values of CCS (above 274.9 Å^2^), which was in accordance with their higher molecular mass and *m*/*z* ratio (above 785.4) (Figure S2B). However, no trendline was obtained from the polycyclic ether and for the amino acid toxin groups due to sample size issue. For instance, the okadaic acid was the only compound belonging to the polycyclic ether family; thus, obtaining a trendline for a group of compounds from a single compound is impractical. Likewise, only three CCS data were obtained for the nonprotein amino acid toxin group. In addition to the trend observed for alkaloids and cyclic peptides, when the experimental CCS values of all singly charged adducts were plotted as a function of *m*/z, a general trend was observed for all of them regardless the group of toxins to which they belonged (Figure 2). A great correlation between *m*/*z* and CCS (*R*^2^ = 0.9655; *n* = 71) and low dispersion of data points were obtained, according to the linear regression model proposed. The dashed lines represent approximately ±5 and ± 10% from the center of the data (gray and black dashed lines, respectively) as determined by the linear fit of the main trendline (solid line).

The dispersion of CCS values at any *m*/*z* region can be appreciated by the curves of ±5 and ±10%. A remarkably low dispersion was obtained as most adducts fall within the established threshold (81% of them fall within ±5%, and only one adduct, the deprotonated okadaic acid, fall slightly above the ±10% threshold), resulting in an interval of CCS predictability, as previously reported by other authors.^44−46^ This is of particular relevance in nontargeted analysis, in which previously uncharacterized phycotoxins can be detected and their identification can be carried out based on the observed *m*/*z* and CCS prediction.

A relevant point is noteworthy when referring to the linear trend line of CCS values versus *m*/*z*, particularly in positive ESI mode. It can be observed a black point at *m*/*z* 514.7 and CCS 177.5 Å^2^ that lies far below the −10% curve. This value corresponds to the CCS of the doubly protonated molecule of microcystin-RR ([M+2H]^2+^). This fact is in accordance with previously reported works, where multiply charged species have also been found far above the +10% curve.^44^ As explained before, the CCS calibration was carried out considering singly charged ions, so CCS measurements are only applicable as valid reference values to single charged ions. In the case of MC-RR, although the monoprotonated and sodium adducts were also observed, the most predominant adduct found by far in both the analysis of standard solutions and the analysis of the spiked and positive dietary supplement samples was the doubly charged molecule. In positive ESI mode, MCs with two arginine residues (R) appear primarily as doubly charged ions while those with one or no R residue are mostly singly protonated,^47^ which is in accordance with recent works where MC-RR was determined using ESI+.^48^ Hence, the CCS value obtained for the diprotonated molecular ion of MC-RR was represented in the graph taking into account that this value could be considered as a reference in the MC-RR identification when using the Major Mix calibration kit employed here. In that sense, although represented in the graph, the linear curve fit to the CCS-*m*/*z* trendline of cyanotoxins excludes the multiply charged [MC-RR + 2H]^2+^ species from the trendline equation.

#### 3.1.1 Limitations to CCS Measurements of BMAA, DAB, and AEG Isomers

As mentioned above, when measuring the CCS of amino acid isomers in negative ionization mode, the CCS values corresponding to the deprotonated adduct of all isomers were observed. Those [M–H]^−^ adducts, with an exact mass of 117.0670, presented ^TW^CCS_N2_ values of 140.6, 140.7, and 141.9 Å^2^ for BMAA, DAB, and AEG, respectively. These results showed differences between them less than 2%, which correspond to the instrumental variation. To make the separation of the three isomers possible, a higher resolving power instrument such as the cyclic IMS or TIMS-TOF would be necessary. On the other hand, when we refer to positive ionization mode, it was found that, in flow injection analysis, the major adduct for the three amino acid isomers was not the protonated species, but the double sodium with proton loss, [M–H + 2Na]^+^ (*m*/*z* 163.0452) (Figure S3). As far as it is known, this adduct has not been reported before in the literature for these analytes. In fact, the most common adduct formed in positive electrospray ionization mode is generally the protonated species, whose CCS value could not be determined under these experimental conditions and with the employed Vion IMS QTOF instrument. The presence of three broad peaks was observed in the ion mobilogram of each amino acid (Figure S4), each of them corresponding with a different species of the [M–H+2Na]^+^ adduct, and yielding to three major CCS values. These values (Table S5) are very similar for the three amino acids, and because their CCS percent difference is lower than ±2%, they will be aligned in the drift time dimension so they will be processed as the same ions as long as they had not been baseline resolved in the chromatographic dimension. From the obtained results, it can be inferred that the different shapes of the amino acid isomer ions were not intensified by the coordination of two sodium atoms within the molecular structures; thus, no distinction between the target analytes can be done through the CCS measurements. In that sense, computational studies would be of great interest to understand the adduct states. In addition, the next step would be to conduct additional studies with different IMS instrumentation to find more convenient adducts, which may present CCS values significantly distinguishable and whose percentage difference are >±2% allowing the identification of the isomers. In the present work, the integration of IMS in the LC–MS workflow did not increase the detection selectivity for BMAA, DAB, and AEG and CCS cannot be taken as a novel parameter for compound identification between these cyanotoxins. Therefore, according to present results, the chromatographic separation provided by HILIC would be necessary to distinguish and identify BMAA, DAB, and AEG isomers (Figure S1B).

### 3.2 Adduct Effect on CCS Values

**Figure 3:**
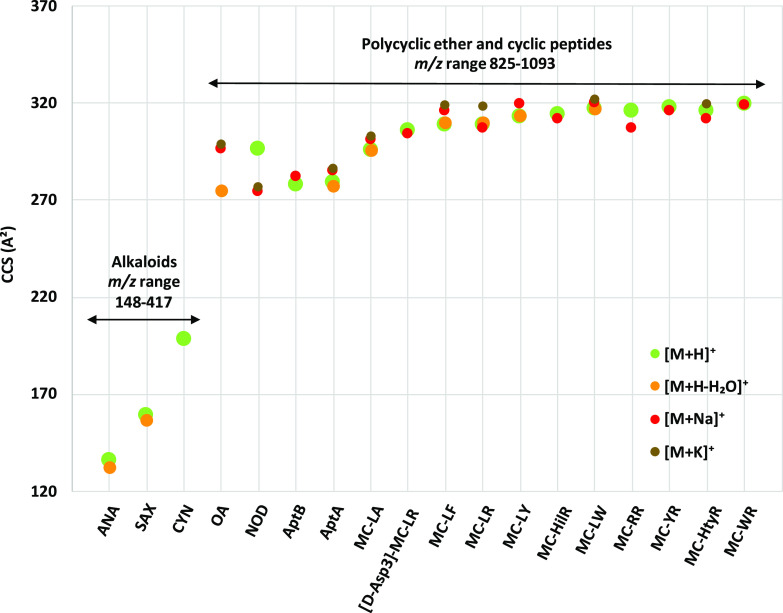
Comparison of experimentally derived CCS in four commonly observed ion adduct states ([M+H]^+^, [M+Na]^+^, [M+K]^+^, and [M+H–H_2_O]^+^).

As previously mentioned, small molecules such as the algal toxins present in this work (118–1067 Da) might form different adducts besides the protonated and deprotonated molecules and they can also form protomers, leading to various ion mobility drift times. As CCS values depend on the charge and on the multiple adduct states, these facts influence the consistency of the developed database for toxin identification in real and complex matrix samples. In fact, different adduct formation can provide additional selectivity and can also show practical utility for identification of analytes and isomers, as not all isomeric structures form the same ionic species in electrospray ionization mode.^49^ For that reason, it is important to include in databases not only the CCS of the most abundant ion formed but also of all the adducts observed. To check the impact of the formed adduct on the CCS values, adducts formed under positive electrospray ionization mode conditions were considered and are displayed in Figure 3.

Generally, sodium and potassium adducts are expected to have higher CCS values than the protonated molecule, as these cations are larger than the proton and consequently the formed adduct interacts more with the nitrogen buffer gas giving rise to a higher drift time value. This trend has been observed in literature when the CCS of molecules such as mycotoxins^28^ or steroids^45^ have been investigated. The difference observed between CCS values of protonated and cationated toxins shows the influence in the structural properties of the adducts in comparison to the protonated molecules. Alkaloids such as ANA, CYN, and SAX did not tend to form sodium or potassium adducts, but they formed the dehydrated adduct (except for CYN) in positive electrospray ionization mode. However, only ANA showed a difference between this CCS and that from the protonated adduct slightly higher than the 2% threshold. For the polycyclic ether marine biotoxin OA, the sodium, potassium, and dehydrated adducts were detected, showing a difference in CCS values between the cationated adducts and dehydrated adduct far higher than 2%. In that case, the protonated molecule was not observed. In fact, its most intense adduct was the deprotonated molecule observed in negative electrospray ionization mode.

On the other hand, all cyclic peptide cyanotoxins (microcystins, anabaenopeptins, and nodularin) leaded to the formation of the sodium adduct apart from the protonated molecule, but only some of them tended to form the potassium adduct and/or dehydrated adduct (see Table 1). In addition, as explained above, only MC-RR leads to the formation of the double-charged [M+2H]^2+^ adduct. For most of the compounds, it was observed that Ω(K^+^) > Ω(H^+^), which is in line with the increase in the size of the potassium cation compared with the proton. Furthermore, the differences CCS values observed between potassium and protonated adducts were higher than 2% for all cyanotoxins excepting MC-LW and MC-HtyR. However, when referring to sodium adducts, there was no consistent trend in the CCS values obtained as it was expected due to the increase in the radii of the ionic metal compared with the proton. Instead, the CCS values of the sodium adducts were quite similar to the protonated molecules and only the sodium adducts of NOD, APa, MC-LF, MC-LY, and MC-RR showed CCS differences greater than 2%. The most remarkable case is nodularin, in which it was observed that both the potassium and sodium adducts presented CCS values well below the protonated molecule, contrary to what was expected. This can be explained by an increased ion compaction and stability after cation binding. However, the CCS differences were higher than 6% for both adducts when compared with the protonated nodularin. Similarly, the dehydrated adducts lead to a decrease in the size of the molecule, so the CCS values are generally lower than those obtained for the protonated molecule Ω(−H_2_O+H^+^) < Ω(H^+^). CCS differences higher than 2% was observed only in the anatoxin alkaloid; however, for all other toxins, the CCS values of the protonated and dehydrated adducts are not significantly different, i.e., less than 2%.

### 3.3 CCS Prediction

The experimentally derived ^TW^CCS_N2_ can also be compared with corresponding predicted values. In this work, CCS values were predicted by different machine learning online tools such as AllCCS, CCSbase, and MetCCS. The achievement of good predictions of CCS values would allow a greater reliability in the identification process, as new phycotoxins can emerge and be characterized by matching predicted and experimental CCS values, despite the lack of analytical standards. Overall, 61, 59, and 60 ions (anions plus cations) were considered for AllCCS, CCSbase, and MetCCS, respectively. Considering protonated, deprotonated, potassium, sodium, and dehydrated adducts, the differences among the machine learning tools lie in the fact that AllCCS does not predict potassium adducts, CCSbase does not predict the dehydrated adducts but it does predict potassium adducts, and MetCCS do not predict neither potassium adducts nor dehydrated adducts in negative mode.

Values predicted by the machine learning approaches showed a Pearson correlation coefficient higher than 0.9220. Despite the great power of these tools, large deviation was observed. For instance, prediction errors were observed within ±2% only for 16, 17, and 7% of the ions when dealing with AllCCS, CCSbase, and MetCCS prediction models, respectively, regardless the phycotoxin species considered. It is remarkable that high percent deviations were found for the predicted values of CCS in negative ESI mode, especially for AllCCS and CCSbase prediction models, where only 5% of the predicted adducts showed a difference within ±2% with respect to the experimental value. Results of predicted CCS values and percent differences compared to the experimental values can be observed in Figure S5 and Table S6. One of the possible reasons for these high errors might be that the CCS data used to develop the training set were ^DT^CCS_N2_ employing the stepped field method.^50^ Thus, our results seem to suggest that it would be necessary a training set composed by ^TW^CCS_N2_ to obtain more accurate results. According to results previously reported by Righetti et al., it was observed that the CCSbase prediction model provided more accurate CCS values, as it includes measurements on TWIM platforms.^51^ Despite this, it can be concluded that the predictive models are not yet completely accurate for every molecule and formed adducts, thus making necessary the employment of the same class of chemical compounds and the same IMS technology.

### 3.4 CCS Measurements in Blue-Green Algae Samples

Once the ^TW^CCS_N2_ values were obtained in standard solutions, an extract of BGA dietary supplement free of toxins was spiked with a mixture of toxins and analyzed to evaluate the influence of the matrix on CCS measurements. The robustness of CCS measurements was carried out by comparing the average ^TW^CCS_N2_ values obtained with standard solutions with those obtained with spiked blue-green algae. For that purpose, dietary supplements were treated, spiked with a mixture of the phycotoxins at 500 μg/L, and analyzed following the procedure detailed in the Experimental Section 2.3. As a proof of concept, the toxins investigated in this study were the ones analyzed by the reversed phase method (i.e., all of them except anatoxin and the amino acid isomers), and the selected concentration level ensured that the peak intensity was high enough to be detected under both positive and negative ESI modes. In addition to spiked samples, blanks of BGA dietary supplements were also analyzed.

**Figure 4:**
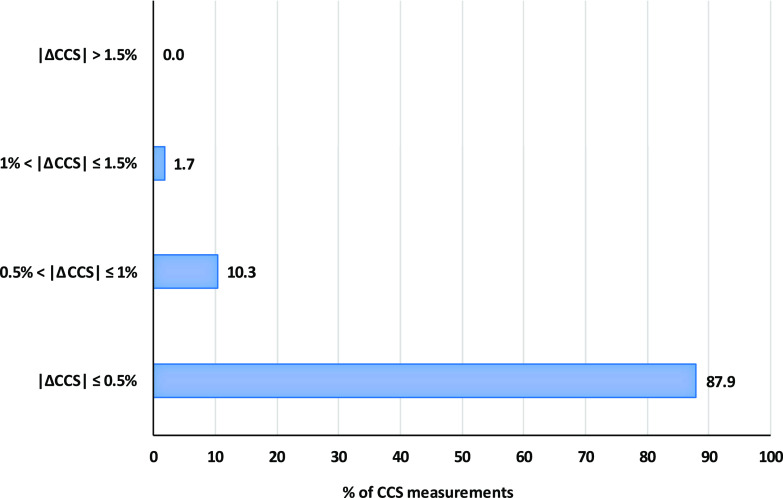
Accuracy of CCS measurements of cyanotoxins in BGA-derived dietary supplements.

Among all CCS values measured in BGA samples, high accuracy and robustness was generally achieved when compared with those derived in pure solvent. As can be seen in Figure 4, more than 87% of the CCS values obtained in the matrix showed a deviation lower than 0.5% with respect to the standard solutions measurements, around 10% of the values presented an error between 0.5 and 1%, and only one measurement, corresponding with the potassium adduct of NOD, showed an error higher than 1%, which was in any case lower than the established threshold value of 2%. The small differences in CCS values between standards and spiked BGA extracts, ranging from 0.0 to 1.3%, proved the reliability in CCS measurements.

### 3.5 Application of Ion Mobility-Derived Information to the Analysis of BGA-Derived Dietary Supplements

As stated above, the implementation of IMS in LC-HRMS workflows enhances the detection of compounds in both targeted and nontargeted analyses because CCS provides an additional parameter for compound identification, which may enhance sensitivity and selectivity. In this sense, the proposed UHPLC-TWIMS-QTOF method was applied to the analysis of dietary supplements containing BGA to demonstrate for the first time the applicability of the IMS technology for the determination of phycotoxins in BGA dietary supplements. As a proof of concept, 19 samples based on diverse BGA (listed in Table S2) from different markets were analyzed applying the reversed phase UHPLC-TWIMS-QTOF workflow in a suspect screening approach.

**Figure 5:**
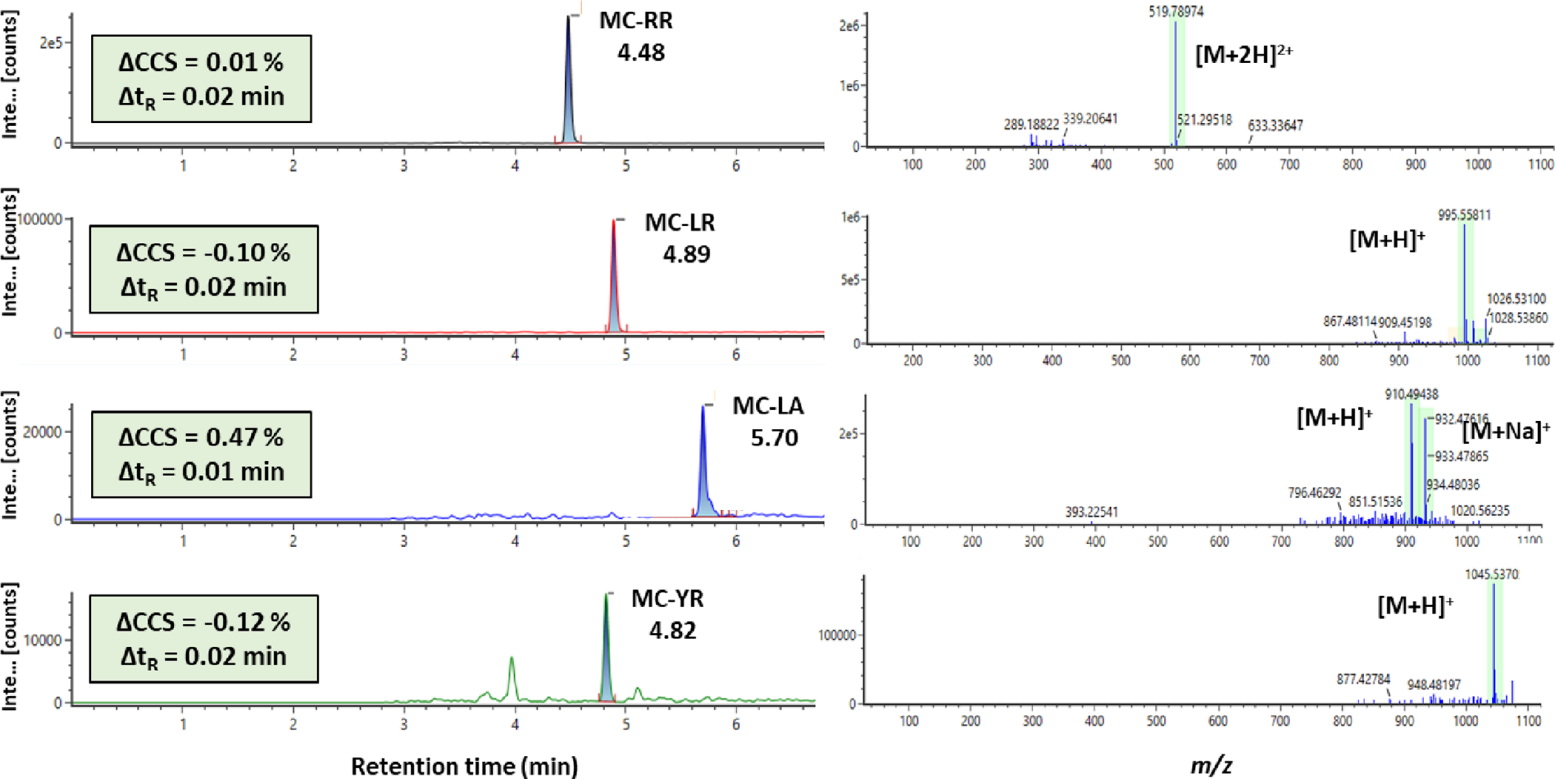
Extracted ion chromatograms and low energy mass spectra of cyanotoxins found in one of the positive BGA dietary supplement samples.

The results showed that 5 out of 19 analyzed samples were positive for at least one cyanotoxin. Overall, five different cyanotoxins were identified in the analyzed samples as they matched retention time, accurate *m*/*z*, and ^TW^CCS_N2_ values with the ones obtained from the standard solutions. MC-LA was positively identified in 4 out of the 5 positive samples. In this line, earlier studies carried out in algal dietary supplements from the Belgium market have also reported this MC as one of the most frequently detected.^52^ MC-LR and MC-RR were found in two samples, while MC-YR and APb were identified in one sample. In this sense, one of the analyzed BGA dietary supplements contained up to four different MCs. Figure 5 shows the extracted ion chromatogram and low mass spectra of each cyanotoxin found in that sample. It has also been specified the Δ*t*_R_ value, which is the difference between the retention time observed in the positive sample and the retention time observed in standard solution, and the ΔCCS value, which represents the difference of the CCS value observed in the positive dietary supplement sample compared with the experimental CCS value obtained in standard solutions, following eq 1.
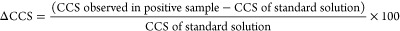
1As it can be observed, the ΔCCS values are below the threshold of 2%, so it is verified that, indeed, the CCS can be taken as an additional identification point. Moreover, these results are in line with those previously reported by other authors, where MCs were not present in spirulina samples, but they were almost exclusively detected in products containing *Aphanizomenon flos-aquae.*(15,18,52−55)

The comprehensive ^TW^CCS_N2_ database developed provided reliable and reproducible *m*/*z* values, retention times, and ^TW^CCS_N2_ values for 81 adducts (including ESI^+^ and ESI^–^ modes), extending the limited information currently available about the CCS characterization of natural toxins. The reliability and robustness of the ^TW^CCS_N2_ measurements were also demonstrated, as their values were constant and independent from the sample matrix (87% of the CCS values obtained in the spiked matrix showed a deviation less than 0.5% with respect to the standard solutions measurements used to obtain the database). Nevertheless, further studies would be highly recommended to extend this investigation and verify CCS measurements in an interlaboratory study and among different IMS systems, such as DTIMS, TIMS, or differential mobility analyzers. Despite the trend observed regardless of the toxin classes, the characterization of a larger number of compounds for each group would be very useful to identify the structural family distribution trends more clearly. Moreover, it would improve the reliability of identification of unknown substances in IMS-MS by determining the chemical categories in complex samples. In addition, while the separation in the drift time dimension of the critical trio of cyanotoxin isomers was addressed, further computational and experimental studies would be advisable to achieve significantly different CCS values that could be used as identification parameters. As a proof of concept, the applicability of this approach was evaluated in the screening of cyanotoxins by analyzing various commercial BGA dietary supplements. Several positive samples were found, being MC-LA the most frequently detected toxin, confirming the need to further investigate the occurrence of such toxins in food supplements to provide data for risk assessment. The obtained CCS values in positive-analyzed samples exhibited small percent deviations (ΔCCS < 2%) compared with database, which verifies CCS as an additional identification parameter, adding confidence in cyanotoxin identification. The availability of this approach is relevant also from the perspective of the expected and ongoing increase in applications for novel food registrations based on cyanobacteria and algae that will sustain the need for punctual, reliable, and flexible analytical approaches to cyanotoxin analysis.

## Supporting Information Available

Toxicity, structure, and physico-chemical properties of target toxins; list of analyzed BGA dietary supplements; composition of CCS calibration solution used for positive ionization mode; composition of CCS calibration solution used for negative ionization mode; CCS values obtained for BMAA, DAB, and AEG isomers using N_2_ as drift gas; theoretical CCS of target toxins obtained using AllCCS, CCSbase, and MetCCS online tools; chromatographic separation of toxins using reversed phase and HILIC methods; correlations of *m*/*z* and measured TWIMS-derived CCS values of alkaloid and cyclic peptide groups of cyanotoxins; adducts observed using flow injection analysis for the nonprotein amino acids; mobilograms obtained from the [M–H + 2Na]^+^ adducts of the nonprotein amino acid isomers AEG, DAB, and BMAA; predicted vs experimentally observed ^TW^CCS_N2_ values obtained with AllCCS, CCSbase, and MetCCS online tools; and spread of CCS percent deviations (ΔCCS) according to most abundant adduct ions monitored with AllCCS, CCSbase, and MetCCS (PDF)
