# Supplementary material for: Implementing the Use of Collision Cross Section Database for Phycotoxin Screening Analysis
Source: J Agric Food Chem. 2023 Jun 22;71(26):10178–89. doi: 10.1021/acs.jafc.3c01060 (PMC10326910; doi:10.1021/acs.jafc.3c01060)
Supplement: Supplementary file 1 — jf3c01060_si_001.pdf [file jf3c01060_si_001.pdf]

Supporting information for:

Implementing the use of collision cross section database for phycotoxin screening analysis

M. Mar Aparicio-Muriana [a,b*], Renato Bruni [a], Francisco J. Lara [b], Monsalud del Olmo-Iruela [b], Maykel Hernandez-Mesa [b], Ana M. Garcia-Campana [b], Chiara Dall'Asta [a], Laura Righetti [a, c, d*]

[a] Department of Food and Drug, University of Parma, Parco Area delle Scienze 17/A, 43124 Parma, Italy

[b] Department of Analytical Chemistry, Faculty of Sciences, University of Granada, Campus Fuentenueva s/n, 18071 Granada, Spain

[c] Laboratory of Organic Chemistry, Wageningen University, 6708, WE, Wageningen, the Netherlands

[d] Wageningen Food Safety Research, Wageningen University & Research, 6700, AE, Wageningen, the Netherlands

Table S1. Toxicity, structure and physico- chemical properties of target toxins.

Toxin group	Toxicity	Name	Formula	Molecular weight	Purity	Seller	Chemical structure
Cyclic peptides	Hepatotoxin	Microcystin-Leucine-Arginine (MC-LR)	$C_{49}H_{74}N_{10}O_{12}$	994.5488	≥99%	Enzo Life Sciences, Inc. (Lausen, Switzerland)	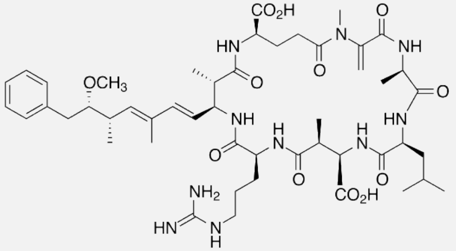
		Microcystin-Arginine-Arginine (MC-RR)	$C_{49}H_{75}N_{13}O_{12}$	1037.5658	≥99%	Enzo Life Sciences, Inc. (Lausen, Switzerland)	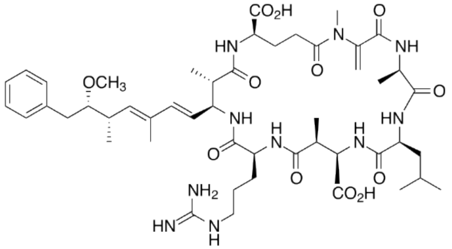
		Microcystin-Tryptophan-Arginine (MC-WR)	$C_{54}H_{73}N_{11}O_{12}$	1067.5440	≥99%	Enzo Life Sciences, Inc. (Lausen, Switzerland)	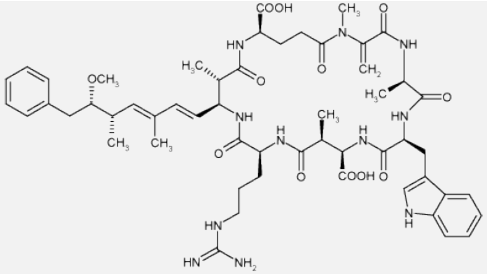

	Microcystin-Homoisoleucine-Arginine (MC-HilR)	$C_{50}H_{76}N_{10}O_{12}$	1008.5644	≥99%	Enzo Life Sciences, Inc. (Lausen, Switzerland)	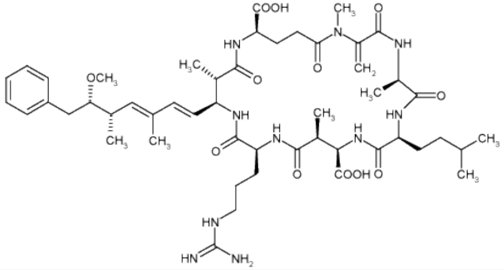
	Microcystin-Homotyrosine-Arginine (MC-HtyR)	$C_{53}H_{74}N_{10}O_{13}$	1058.5437	≥99%	Enzo Life Sciences, Inc. (Lausen, Switzerland)	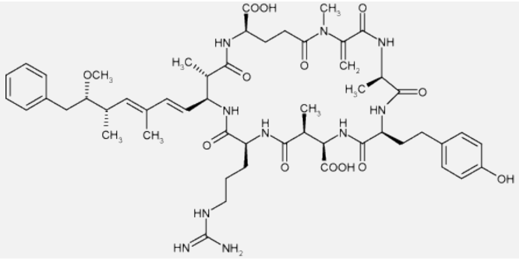
	Microcystin-Tyrosine-Arginine (MC-YR)	$C_{52}H_{72}N_{10}O_{13}$	1044.5280	≥99%	Enzo Life Sciences, Inc. (Lausen, Switzerland)	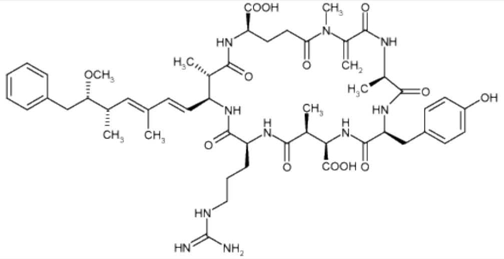
	Microcystin-Leucine-Tryptophan (MC-LW)	$C_{54}H_{72}N_{8}O_{12}$	1024.5270	≥99%	Enzo Life Sciences, Inc. (Lausen, Switzerland)	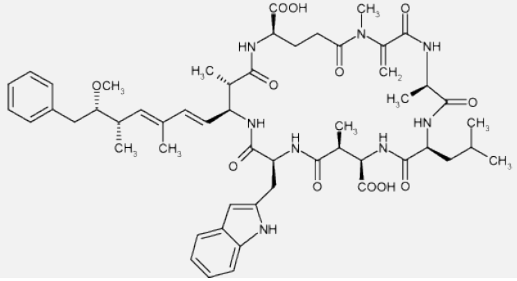

	Microcystin-Leucine-Tyrosine (MC-LY)	$C_{52}H_{71}N_7O_{13}$	1001.5110	≥99%	Enzo Life Sciences, Inc. (Lausen, Switzerland)	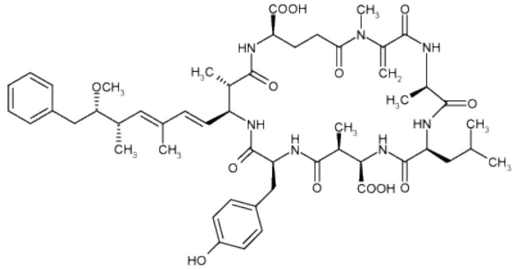
	[D-Asp3]Microcystin-Leucine-Arginine ([D-Asp3]-MC-LR)	$C_{48}H_{72}N_{10}O_{12}$	980.5331	≥99%	Enzo Life Sciences, Inc. (Lausen, Switzerland)	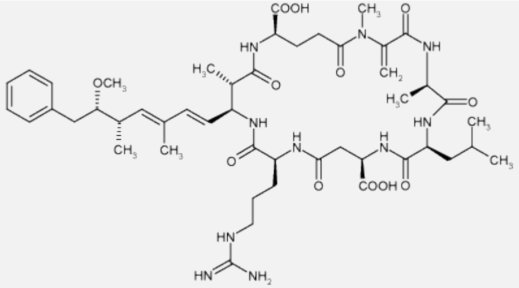
	Microcystin-Leucine-Phenilalanine (MC-LF)	$C_{52}H_{71}N_7O_{12}$	985.5161	≥99%	Enzo Life Sciences, Inc. (Lausen, Switzerland)	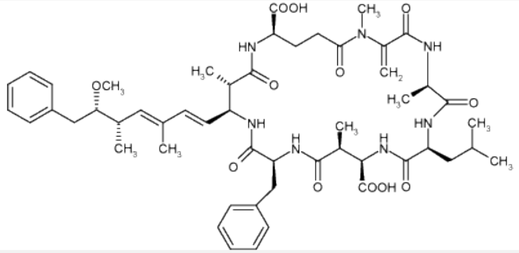
	Microcystin-Leucine-Alanine (MC-LA)	$C_{46}H_{67}N_7O_{12}$	909.4848	≥99%	Enzo Life Sciences, Inc. (Lausen, Switzerland)	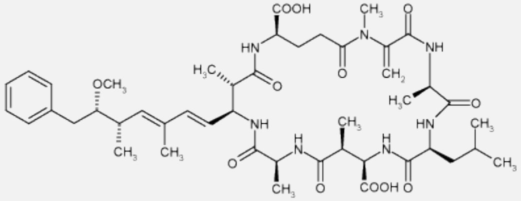

Alkaloids	Cytotoxin	Anabaenopeptin A (APa)	$C_{44}H_{57}N_7O_{10}$	843.4167	≥95%	Enzo Life Sciences, Inc. (Lausen, Switzerland)	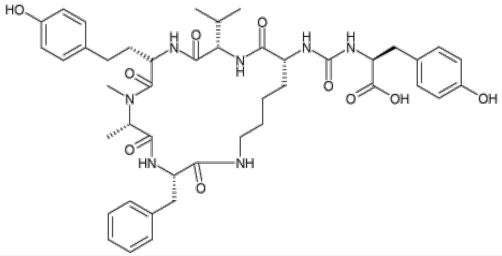
		Anabaenopeptin B (APb)	$C_{41}H_{60}N_{10}O_9$	836.4545	≥95%	Enzo Life Sciences, Inc. (Lausen, Switzerland)	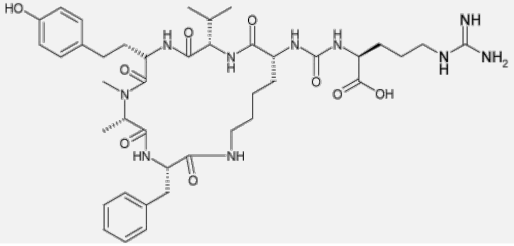
		Nodularin (NOD)	$C_{41}H_{60}N_8O_{10}$	824.4432	≥95%	Enzo Life Sciences, Inc. (Lausen, Switzerland)	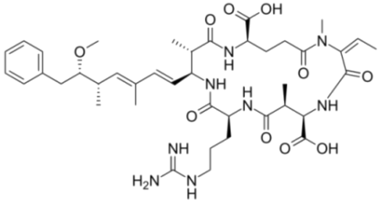
		Cilindrospermopsin (CYN)	$C_{15}H_{21}N_5O_7S$	415.1162	≥95%	Enzo Life Sciences, Inc. (Lausen, Switzerland)	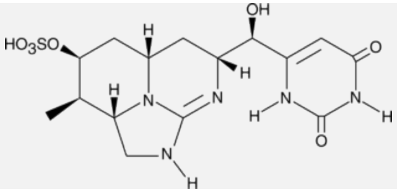
		Saxitoxin (SAX)	$C_{10}H_{17}N_7O_4$	299.1342		Enzo Life Sciences, Inc. (Lausen, Switzerland)	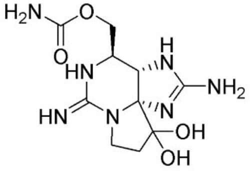

Non-protein amino acids	Neurotoxin	Anatoxin-a (ANA)	$C_{10}H_{15}NO$	165.1154	≥98%	Enzo Life Sciences, Inc. (Lausen, Switzerland)	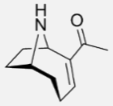
		β-methylamino-L-alanine (BMAA)	$C_4H_{10}N_2O_2$	118.0742	≥97%	Sigma Aldrich (Darmstadt, Germany)	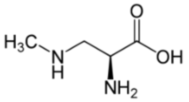
		2,4-diaminobutyric acid (DAB)	$C_4H_{10}N_2O_2$	118.0742	≥95%	Sigma Aldrich (Darmstadt, Germany)	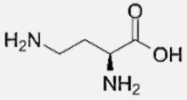
		N-(2-aminoethyl)glycine (AEG)	$C_4H_{10}N_2O_2$	118.0742	≥98%	Sigma Aldrich (Darmstadt, Germany)	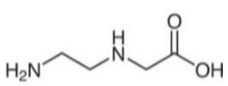
Polycyclic ether	Neurotoxin	Okadaic Acid (OA)	$C_{44}H_{68}O_{13}$	804.4660	≥95%	Cayman Chemicals (Michigan, USA)	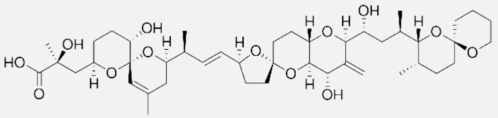

Table S2. List of analyzed BGA dietary supplements.

Sample	Acquired in	Supplier	Origin	Form	Composition	Daily dose
**1**	Spain	Local store (Granada)	Costa Rica	Powder	Pure spirulina (*Arthrospira platensis*)	6 g product, i.e, 6000 mg spirulina
**2**	Spain	Online	USA	Capsule	Spirulina (*Arthrospira platensis* Gomont), hydroxypropyl methylcellulose, chlorella (*Chlorella vulgaris* Beijerinck), fucus (*Fucus vesiculosus* L.), vitamin C, magnesium stearate	6 capsules, i.e, 891 mg spirulina, 445.5 mg chlorella, 445.5 mg fucus
**3**	Spain	Online	Spain	Tablet	Spirulina (*Arthrospira platensis*), chlorella (*Chlorella vulgaris),* ferrous fumarate, folic acid, vitamin B12	3 capsules, i.e, 750 mg spirulina, 750 mg chlorella
**4**	Spain	Online	Spain	Tablet	Klamath (*Aphanizomenon flos-aquae*), spirulina (*Spirulina platensis* Geitler), microcrystalline cellulose, silicon dioxide	3 capsules, i.e, 600 mg klamath (Aphanizomenon flos aquae), 600 mg spirulina
**5**	Spain	Online	Agriculture non-UE	Tablet	Chlorella (*Chlorella pyrenoidosa* Chick), silicon dioxide	8 capsules, i.e, 3268 mg chlorella
**6**	Spain	Online	Agriculture non-UE	Tablet	Spirulina (*Spirulina platensis* Geitler), dioxide de silício	6 capsules, i.e, 2562 mg spirulina
**7**	Spain	Local Store (Granada)	Unknown	Capsule	Spirulina (*Arthrospira platensis*), hydroxypropyl methylcellulose	6 capsules, i.e, 2160 mg spirulina

**8**	Spain	Local Store (Granada)	Unknown	Powder	Pure Spirulina (*Arthrospira platensis*)	Not available
**9**	Spain	Local store	Unknown	Powder	Pure Klamath (*Aphanizomenon flos-aquae*)	Not available
**10**	Italy	Online	Agriculture non-UE	Tablet	Pure Klamath (*Aphanizomenon flos-aquae*)	3 capsules, i.e, 1500 mg klamath
**11**	Italy	Online	Agriculture non-UE	Tablet	Klamath (*Aphanizomenon flos-aquae*) 99.5%, anti-caking: talc 0.5%	4 capsules, i.e, 1990 mg kalmath
**12**	Italy	Online	USA	Capsule	Klamath (*Aphanizomenon flos-aquae*), vegetal capsule: hydroxipropyl methylcellulose, potato starch	6 capsules, i.e, 2400 mg klamath
**13**	Italy	Online	France	Liquid	Klamath (*Aphanizomenon flos-aquaea*) 40%, *Spirulina platensis* extract (phycocyanin) 50%, stabilizer: glycerol, Flavour: tea tree essential oil	5 mL product, i.e., 2 mL klamath
**14**	Italy	Online	Italy	Tablet	Klamath (*Aphanizomenon flos-aquae*), adjuvants: hydroxypropyl cellulose, silicium dioxide, microcrystalline cellulose, magnesium stearate, vegetal capsule; hydroxipropyl methylcellulose, polyethylenglicol	3 capsules, i.e, 2700 mg klamath
**15**	Italy	Online	USA	Powder	Pure klamath (*Aphanizomenon flos-aquae*)	1 g product, i.e., 1000 mg klamath
**16**	Italy	Local Store (Parma)	Italy	Tablet	*Spirulina platensis*, anti-caking agent: talc	6 capsules, i.e, 2985 mg spirulina

**17**	Italy	Online	Italy	Capsule	Pure *Spirulina platensis*	6 capsules, i.e, 2400 mg spirulina
**18**	Italy	Online	Italy	Capsule	Chlorella (*Chlorella pyrenoidosa* H. Chick), inlunin, anti-caking agent: magnesium salt from fatty acid, vegetal capsule (hydroxipropyl methylcellulose)	4 capsules, i.e, 1400 mg chlorella and 170 mg inulin
**19**	Italy	Online	USA	Powder	Pure Klamath (*Aphanizomenon flos-aquae*)	2 g product, i.e., 2000 mg klamath

Table S3. Composition of CCS calibration solution used for positive ionization mode.

Compound	*m/z*	CCS (Å^2)
**Acetaminophen**	152.0706	130.4
**Reserpine fragment**	195.0877	138.2
**Sulfaguanidine**	215.0597	146.8
**Sulfadimethoxine**	311.0809	168.4
**Val-Tyr-Val**	380.2180	191.7
**Verapamil**	455.2904	208.8
**Terfenadine**	472.3210	228.7
**Polyalanine**	516.2776	211.0
**Leucine Enkephalin**	556.2766	229.8
**Polyalanine**	587.3148	252.3
**Reserpine**	609.2807	252.3
**Polyalanine**	658.3519	243.0
**Polyalanine**	729.3890	256.0
**Polyalanine**	800.4261	271.0
**Polyalanine**	871.4632	282.0
**Polyalanine**	942.5003	294.0
**Polyalanine**	1013.5374	306.0
**Polyalanine**	1084.5746	321.5
**Polyalanine**	1155.6117	333.6
**Ultramark 1621**	1022.0034	263.1
**Ultramark 1621**	1121.9970	276.5
**Ultramark 1621**	1221.9843	291.2
**Ultramark 1621**	1321.9843	304.0
**Ultramark 1621**	1421.9779	316.7
**Ultramark 1621**	1521.9715	329.0
**Ultramark 1621**	1621.9651	340.1
**Ultramark 1621**	1721.9587	351.3
**Ultramark 1621**	1821.9523	362.1
**Ultramark 1621**	1921.9459	372.6

Table S4. Composition of CCS calibration solution used for negative ionization mode.

Compound	m/z	CCS (Å^2)
**Acetaminophen**	150.0561	131.5
**Theophylline**	179.0575	132.4
**Sulfaguanidine**	213.0452	145.2
**Sulfadimethoxine**	309.2034	170.1
**Val-Tyr-Val**	378.2034	192.5
**Leucine Enkephalin**	554.2620	225.3
**Perfluoroheptanoic acid-CO2**	318.9766	130.1
**Perfluoroheptanoic acid-CO2**	368.9766	137.2
**Polyalanine**	585.3002	227.7
**Reserpine**	607.2661	265.2
**Polyalanine**	656.3373	242.1
**Polyalanine**	727.3744	255.9
**Polyalanine**	798.4115	268.5
**Polyalanine**	869.4487	280.2
**Polyalanine**	940.4856	294.6
**Polyalanine**	1011.5288	308.8
**Polyalanine**	1082.5600	322.4
**Ultramark 1621**	1165.9880	275.8
**Ultramark 1621**	1265.9816	288.0
**Ultramark 1621**	1365.9752	299.7

**Ultramark 1621**	1465.9688	311.7
**Ultramark 1621**	1565.9624	323.7
**Ultramark 1621**	1665.9560	334.7
**Ultramark 1621**	1765.9496	346.2
**Ultramark 1621**	1865.9432	357.1
**Ultramark 1621**	1965.9369	367.2

Table S5. CCS values obtained for BMAA, DAB and AEG isomers using N_2 as drift gas.

**Compound**	**Adduct**	**Theoretical exact *m/z***	**Experimental CCS**	**SD**	**RSD (%)**
β-methylamine-L-alanine	$[M\text{-}H+2Na]^+$	163.0459	**133.4**	0.75	0.56
β-methylamine-L-alanine	$[M\text{-}H+2Na]^+$	163.0459	**161.8**	0.80	0.50
β-methylamine-L-alanine	$[M\text{-}H+2Na]^+$	163.0459	**182.7**	2.72	1.49
2,4-diaminobutyric acid	$[M\text{-}H+2Na]^+$	163.0459	**132.7**	-	-
2,4-diaminobutyric acid	$[M\text{-}H+2Na]^+$	163.0459	**159.4**	0.91	0.57
2,4-diaminobutyric acid	$[M\text{-}H+2Na]^+$	163.0459	**177.9**	4.66	2.62
N-(2-aminoethyl)glycine	$[M\text{-}H+2Na]^+$	163.0459	**132.4**	0.30	0.23
N-(2-aminoethyl)glycine	$[M\text{-}H+2Na]^+$	163.0459	**164.2**	0.83	0.50
N-(2-aminoethyl)glycine	$[M\text{-}H+2Na]^+$	163.0459	**185.3**	1.13	0.61

Table S6. Theoretical CCS of target toxins obtained using AllCCS, CCSbase and MetCCS online tools.

Compound	Adduct	Experimental CCS	AllCCS prediction	ΔCCS (%) AllCCS	CCSbase prediction	ΔCCS (%) CCSBase	MetCCS prediction	ΔCCS (%) MetCCS
**β-methylamine-L-alanine**	[M-H]-	**140.6**	124.4	-13.0	123.5	-13.9	123.4	-14.0
**2,4-diaminobutyric acid**	[M-H]-	**140.7**	121.8	-15.6	120.9	-16.4	119.8	-17.5
**N-(2-aminoethyl)glycine**	[M-H]-	**141.9**	125.1	-13.4	122.8	-15.5	116.4	-21.9
**Anatoxin-a**	$[M+H]^+$	**136.1**	137.6	1.1	129.5	-5.1	132.9	-2.4
**Anatoxin-a**	$[M+H-H_2O]^+$	**132.6**	133.2	0.4		0.0	128.2	-3.5
**Saxitoxin**	$[M+H]^+$	**159.6**	166.2	3.9	170.9	6.6	173.5	8.0
**Saxitoxin**	$[M+H-H_2O]^+$	**157.2**	162.8	3.5			171.0	8.1
**Cylindrospermopsin**	$[M+H]^+$	**198.6**	194.8	-1.9	198.7	0.1	187.0	-6.2
**Cylindrospermopsin**	$[M-H]^-$	**198.9**	191.3	-4.0	194.9	-2.1	186.7	-6.6
**Okadaic acid**	$[M-H]^-$	**308.4**	286.10	-7.8	277.70	-11.1	280.5	-10.0
**Okadaic acid**	$[M+Na]^+$	**296.9**	272.90	-8.8	277.50	-7.0	308.1	3.6
**Okadaic acid**	$[M+K]^+$	**298.9**			284.80	-5.0		
**Okadaic acid**	$[M+H-H_2O]^+$	**275.5**	271.6	-1.4			304.6	9.6
**Nodularin**	$[M+H]^+$	**296.5**	284.3	-4.3	302.2	1.9	277.5	-6.8
**Nodularin**	$[M-H]^-$	**288.8**	268.2	-7.7	303.3	4.8	283.8	-1.8
**Nodularin**	$[M+Na]^+$	**274.9**	284.8	3.4	301.5	8.8	277.9	1.1
**Nodularin**	$[M+K]^+$	**277.3**			273.3	-1.4		
**Anabaenopeptin B**	$[M+H]^+$	**278.3**	283.7	1.9	304.3	8.6	251.5	-10.6
**Anabaenopeptin B**	$[M-H]^-$	**286.4**	263.1	-8.9	303.0	5.5	275.3	-4.0
**Anabaenopeptin B**	$[M+Na]^+$	**282.2**	283.5	0.5	302.5	6.7	252.7	-11.7
**Anabaenopeptin B**	$[M-H-H_2O]^-$	**280.5**	283.8	1.2				
**Anabaenopeptin A**	$[M+H]^+$	**279.2**	284.6	1.9	307.4	9.2	259.2	-7.7
**Anabaenopeptin A**	$[M-H]^-$	**278.3**	247.0	-12.7	305.7	9.0	276.7	-0.6
**Anabaenopeptin A**	$[M+Na]^+$	**285.5**	284.5	-0.4	306.6	6.9	260.8	-9.5
**Anabaenopeptin A**	$[M+K]^+$	**286.7**			288.1	0.5		
**Anabaenopeptin A**	$[M+H-H_2O]^+$	**277.8**	284.7	2.4			257.8	-7.7
**Microcystin-LA**	$[M+H]^+$	**296.0**	304.0	2.6	313.7	5.6	276.5	-7.1

**Microcystin-LA**	$[M-H]^-$	**317.2**	274.6	-15.5	308.6	-2.8	280.6	-13.0
**Microcystin-LA**	$[M+Na]^+$	**301.3**	304.1	0.9	312.0	3.4	277.5	-8.6
**Microcystin-LA**	$[M+K]^+$	**303.2**			292.2	-3.8		
**Microcystin-LA**	$[M+H-H_2O]^+$	**296.0**	303.9	2.6			275.6	-7.4
**[D-Asp3]Microcystin-LR**	$[M+H]^+$	**305.9**	319.80	4.3	323.30	5.4	275.5	-11.0
**[D-Asp3]Microcystin-LR**	$[M-H]^-$	**324.8**	288.10	-12.7	317.40	-2.3	288.3	-12.7
**[D-Asp3]Microcystin-LR**	$[M+Na]^+$	**304.7**	319.60	4.7	320.10	4.8	275.8	-10.5
**Microcystin-LF**	$[M+H]^+$	**309.2**	327.3	5.5	324.2	4.6	286.3	-8.0
**Microcystin-LF**	$[M-H]^-$	**329.7**	269.1	-22.5	321.3	-2.6	287.8	-14.5
**Microcystin-LF**	$[M+Na]^+$	**316.3**	327.4	3.4	324.2	2.4	287.1	-10.2
**Microcystin-LF**	$[M+K]^+$	**319.2**			303.7	-5.1		
**Microcystin-LF**	$[M+H-H_2O]^+$	**310.2**	327.2	5.2			285.6	-8.6
**Microcystin-LR**	$[M+H]^+$	**309.3**	319.8	3.3	321.2	3.7	277.8	-11.3
**Microcystin-LR**	$[M-H]^-$	**326.9**	284.3	-15.0	315.5	-3.6	291.8	-12.0
**Microcystin-LR**	$[M+Na]^+$	**307.3**	319.8	3.9	318.2	3.4	277.9	-10.6
**Microcystin-LR**	$[M+K]^+$	**318.9**			294.4	-8.3		
**Microcystin-LR**	$[M+H-H_2O]^+$	**310.2**	319.8	3.0		0.0	277.7	-11.7
**Microcystin-LY**	$[M+H]^+$	**313.5**	327.4	4.3	324.9	3.5	280.6	-11.7
**Microcystin-LY**	$[M-H]^-$	**326.1**	271.6	-20.1	318.4	-2.4	287.4	-13.5
**Microcystin-LY**	$[M+Na]^+$	**320.0**	327.4	2.3	322.4	0.7	280.8	-14.0
**Microcystin-LY**	$[M+H-H_2O]^+$	**313.8**	327.3	4.2			280.3	-11.9
**Microcystin-HilR**	$[M+H]^+$	**314.7**	324.10	2.9	320.10	1.7	280.9	-12.0
**Microcystin-HilR**	$[M-H]^-$	**331.5**	287.90	-15.2	316.20	-4.8	293.2	-13.1
**Microcystin-HilR**	$[M+Na]^+$	**312.1**	324.10	3.7	316.90	1.5	281.2	-11.0
**Microcystin-LW**	$[M+H]^+$	**317.3**	333.7	4.9	327.0	3.0	285.3	-11.2
**Microcystin-LW**	$[M-H]^-$	**332.1**	265.7	-25.0	314.5	-5.6	308.4	-7.7
**Microcystin-LW**	$[M+Na]^+$	**320.7**	333.6	3.9	322.2	0.5	286.2	-12.1
**Microcystin-LW**	$[M+K]^+$	**322.0**			308.6	-4.3		
**Microcystin-LW**	$[M+H-H_2O]^+$	**317.2**	333.6	4.9			284.5	-11.5
**Microcystin-RR**	$[M+H]^+$	**316.4**	322.5	1.9	322.8	2.0	302.9	-4.5
**Microcystin-RR**	$[M-H]^-$	**327.4**	310.8	-5.3	316.2	-3.5	302.9	-8.1

**Microcystin-RR**	$[M+Na]^+$	**307.4**	322.3	4.6	318.1	3.4	303.1	-1.4
**Microcystin-YR**	$[M+H]^+$	**318.0**	328.7	3.3	328.0	3.1	291.8	-9.0
**Microcystin-YR**	$[M-H]^-$	**322.7**	282.7	-14.2	319.9	-0.9	310.5	-3.9
**Microcystin-YR**	$[M+Na]^+$	**316.6**	328.5	3.6	324.0	2.3	292.3	-8.3
**Microcystin-HtyR**	$[M+H]^+$	**316.4**	332.0	4.7	329.9	4.1	295.0	-7.3
**Microcystin-HtyR**	$[M-H]^-$	**331.2**	282.9	-17.1	321.7	-3.0	313.3	-5.7
**Microcystin-HtyR**	$[M+Na]^+$	**312.0**	331.9	6.0	325.7	4.2	295.4	-5.6
**Microcystin-HtyR**	$[M+K]^+$	**319.7**		0.0	303.7	-5.3		
**Microcystin-WR**	$[M+H]^+$	**320.0**	334.2	4.3	328.1	2.5	290.4	-10.2
**Microcystin-WR**	$[M-H]^-$	**328.2**	272.8	-20.3	314.2	-4.5	311.9	-5.2
**Microcystin-WR**	$[M+Na]^+$	**319.5**	334.1	4.3	322.0	0.8	290.8	-9.9

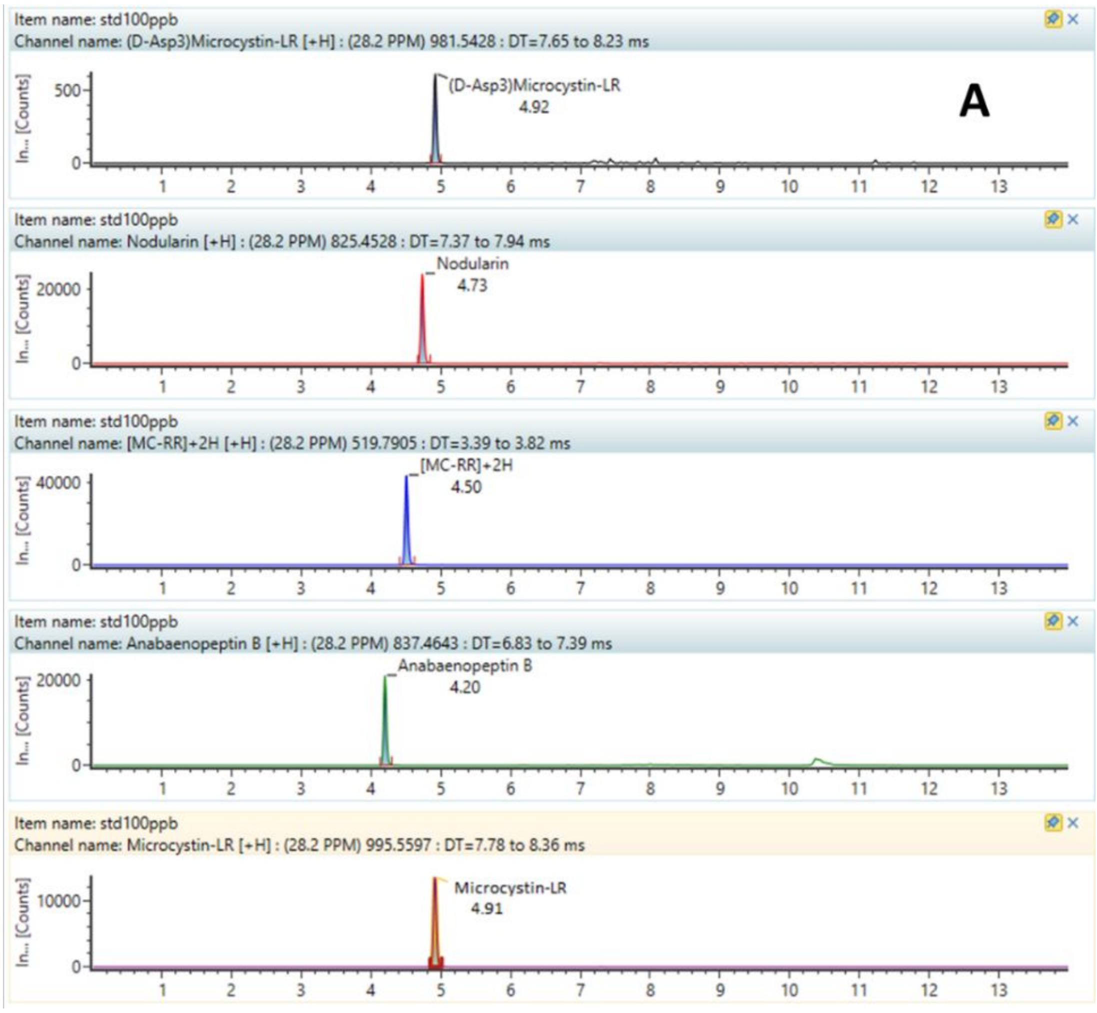

A
Item name: std100ppb
Channel name: (D-Asp3)Microcystin-LR [+H] : (28.2 PPM) 981.5428 : DT=7.65 to 8.23 ms
(D-Asp3)Microcystin-LR
4.92
In... [Counts]
500
0
1
2
3
4
5
6
7
8
9
10
11
12
13
Item name: std100ppb
Channel name: Nodularin [+H] : (28.2 PPM) 825.4528 : DT=7.37 to 7.94 ms
Nodularin
4.73
20000
Item name: std100ppb
Channel name: [MC-RR]+2H [+H] : (28.2 PPM) 519.7905 : DT=3.39 to 3.82 ms
[MC-RR]+2H
4.50
40000
Item name: std100ppb
Channel name: Anabaenopeptin B [+H] : (28.2 PPM) 837.4643 : DT=6.83 to 7.39 ms
Anabaenopeptin B
4.20
20000
Item name: std100ppb
Channel name: Microcystin-LR [+H] : (28.2 PPM) 995.5597 : DT=7.78 to 8.36 ms
Microcystin-LR
4.91
10000

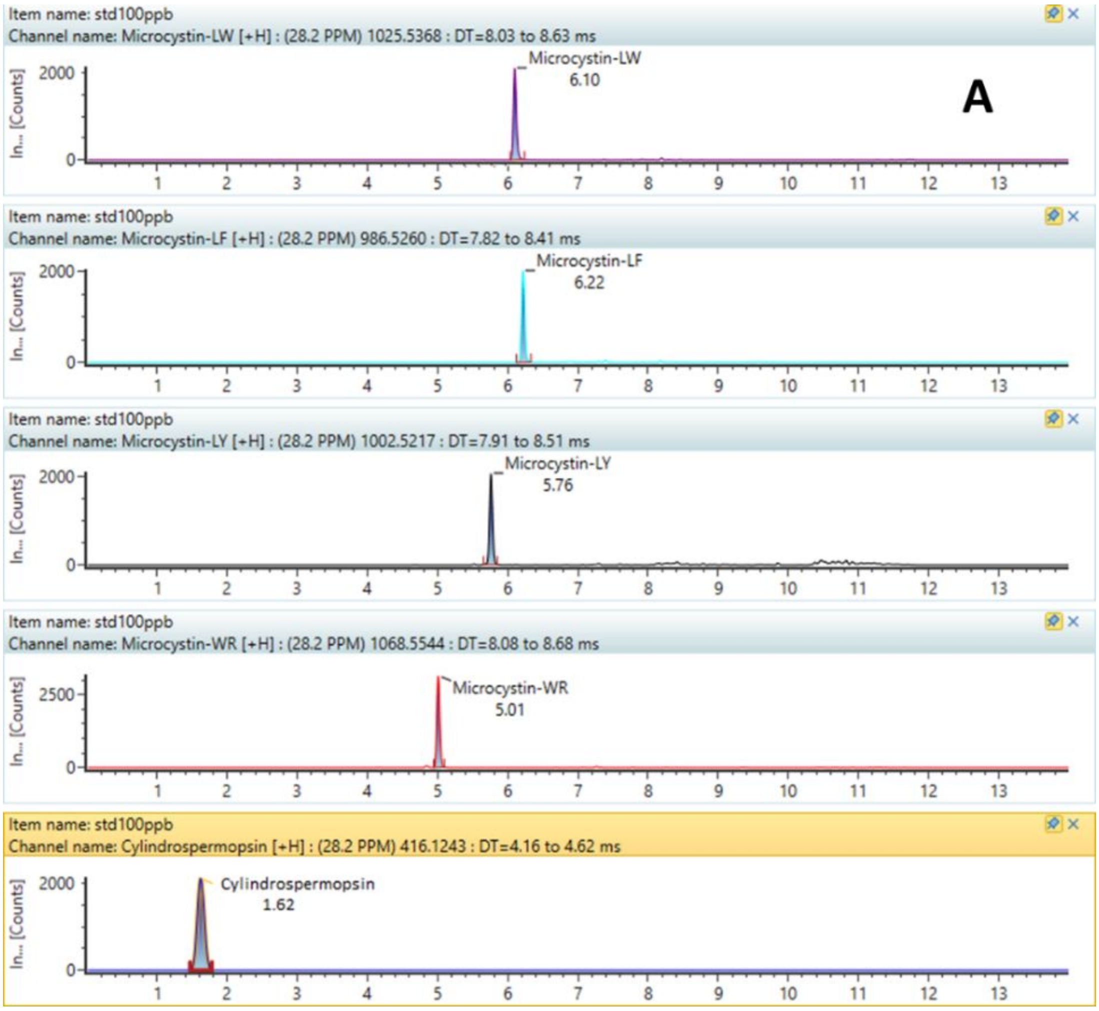

A
Item name: std100ppb
Channel name: Microcystin-LW [+H] : (28.2 PPM) 1025.5368 : DT=8.03 to 8.63 ms
Microcystin-LW
6.10
Item name: std100ppb
Channel name: Microcystin-LF [+H] : (28.2 PPM) 986.5260 : DT=7.82 to 8.41 ms
Microcystin-LF
6.22
Item name: std100ppb
Channel name: Microcystin-LY [+H] : (28.2 PPM) 1002.5217 : DT=7.91 to 8.51 ms
Microcystin-LY
5.76
Item name: std100ppb
Channel name: Microcystin-WR [+H] : (28.2 PPM) 1068.5544 : DT=8.08 to 8.68 ms
Microcystin-WR
5.01
Item name: std100ppb
Channel name: Cylindrospermopsin [+H] : (28.2 PPM) 416.1243 : DT=4.16 to 4.62 ms
Cylindrospermopsin
1.62
In... [Counts]

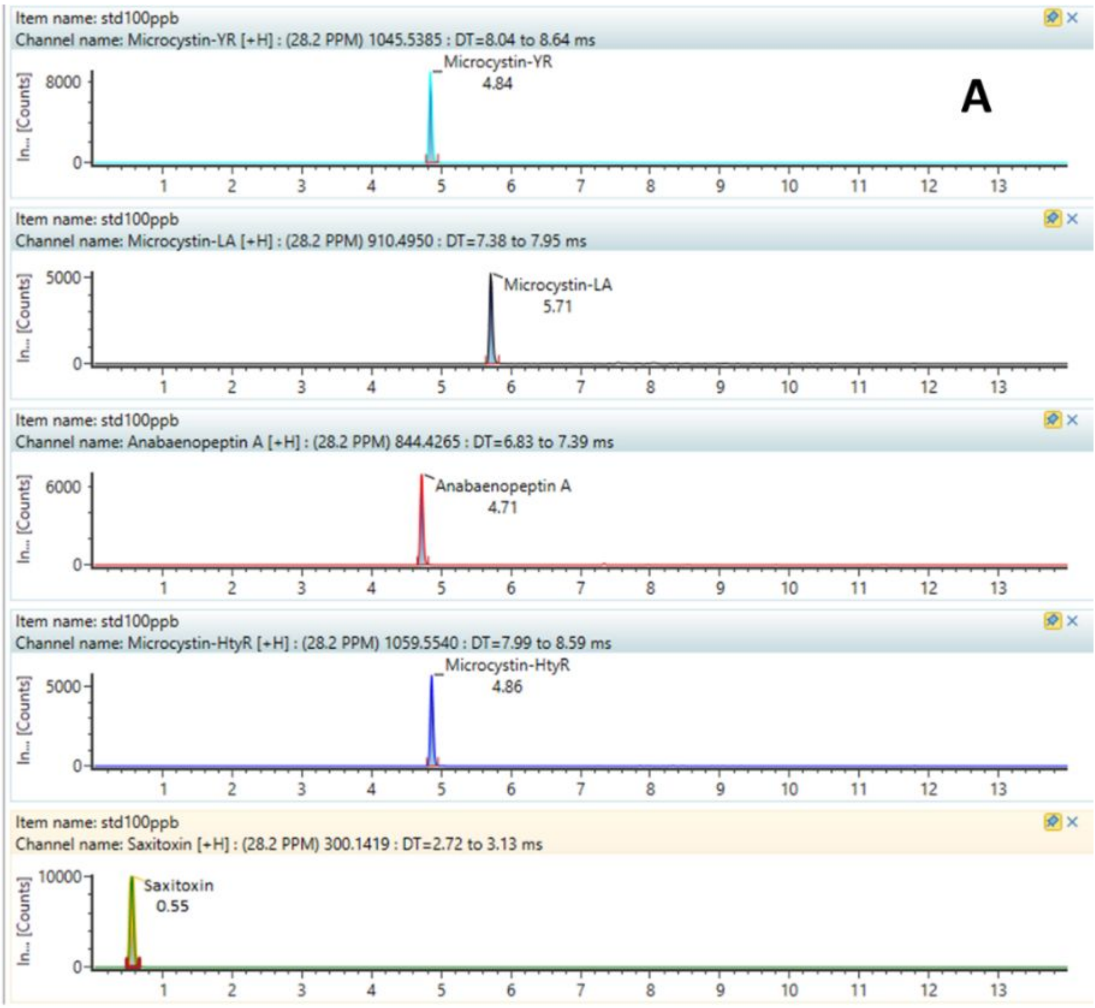

Item name: std100ppb
Channel name: Microcystin-YR [+H] : (28.2 PPM) 1045.5385 : DT=8.04 to 8.64 ms
Microcystin-YR
4.84
A
Item name: std100ppb
Channel name: Microcystin-LA [+H] : (28.2 PPM) 910.4950 : DT=7.38 to 7.95 ms
Microcystin-LA
5.71
Item name: std100ppb
Channel name: Anabaenopeptin A [+H] : (28.2 PPM) 844.4265 : DT=6.83 to 7.39 ms
Anabaenopeptin A
4.71
Item name: std100ppb
Channel name: Microcystin-HtyR [+H] : (28.2 PPM) 1059.5540 : DT=7.99 to 8.59 ms
Microcystin-HtyR
4.86
Item name: std100ppb
Channel name: Saxitoxin [+H] : (28.2 PPM) 300.1419 : DT=2.72 to 3.13 ms
Saxitoxin
0.55
In... [Counts]

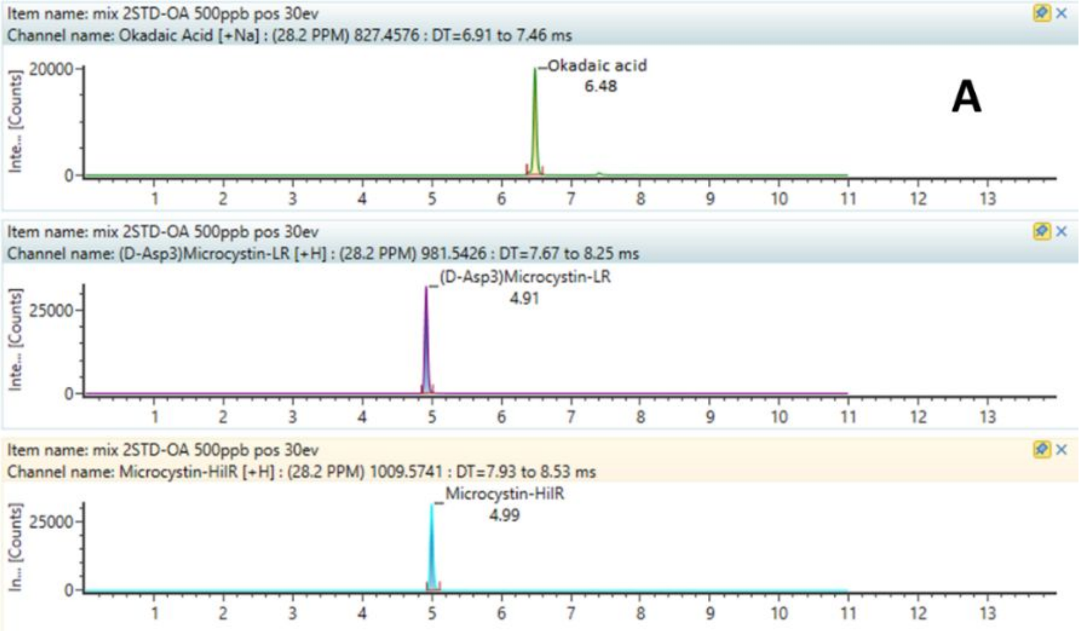

Item name: mix 2STD-OA 500ppb pos 30ev
Channel name: Okadaic Acid [+Na] : (28.2 PPM) 827.4576 : DT=6.91 to 7.46 ms
Okadaic acid
6.48
A
Item name: mix 2STD-OA 500ppb pos 30ev
Channel name: (D-Asp3)Microcystin-LR [+H] : (28.2 PPM) 981.5426 : DT=7.67 to 8.25 ms
(D-Asp3)Microcystin-LR
4.91
Item name: mix 2STD-OA 500ppb pos 30ev
Channel name: Microcystin-HilR [+H] : (28.2 PPM) 1009.5741 : DT=7.93 to 8.53 ms
Microcystin-HilR
4.99
Inte... [Counts]

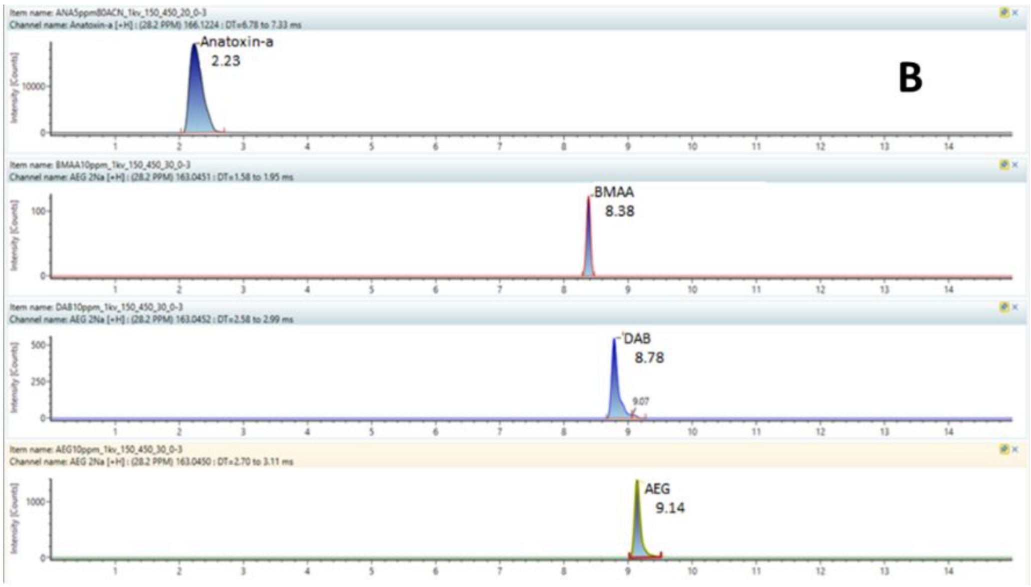

Figure S1. Chromatographic separation of toxins using reversed phase (A) and HILIC (B) methods.

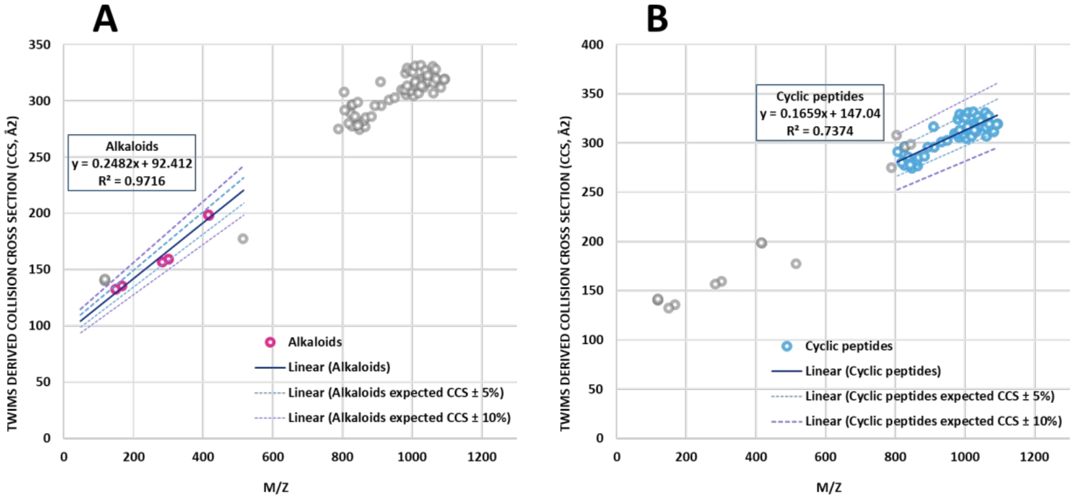

Figure S2. Correlations of *m/z* and measured TWIMS-derived CCS values of A) alkaloid group of cyanotoxins; B) cyclic peptide group of cyanotoxins.

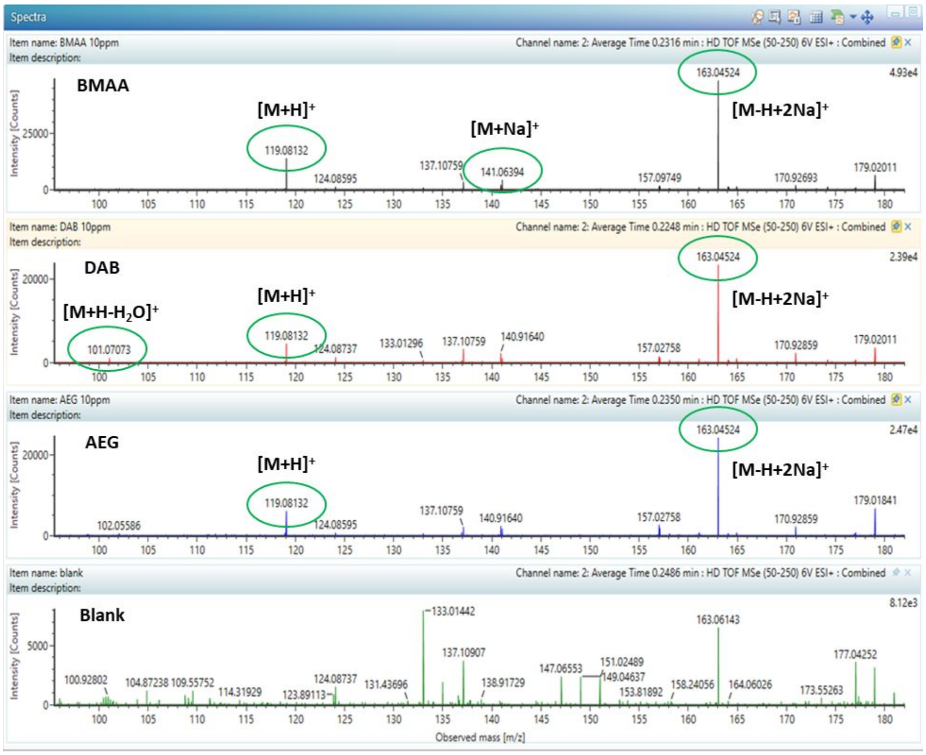

Figure S3. Adducts observed using flow injection analysis for the non-protein amino acids.

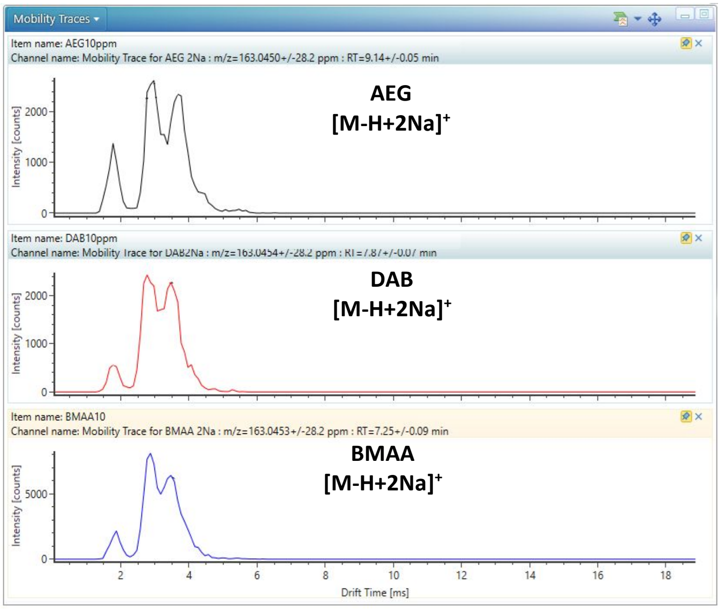

Figure S4. Mobilograms obtained from the $[M-H+2Na]^+$ adducts of the non-protein amino acids isomers AEG, DAB and BMAA.

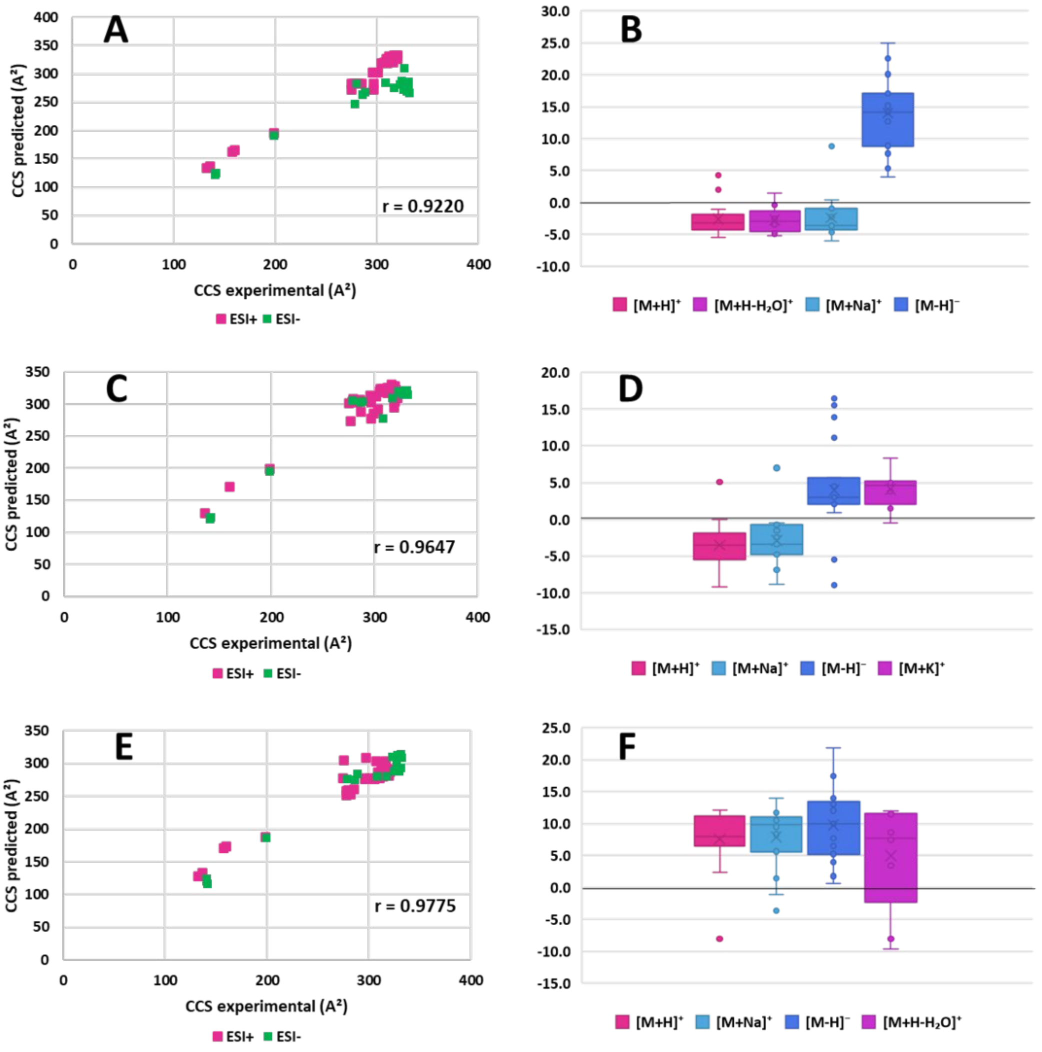

Figure S5. Predicted vs. experimentally observed $^{TW}CCS_{N2}$ values obtained with AllCCS (A), CCSbase (C) and MetCCS (E) online tools (A: Pearson r = 0.9220, $p < 0.001$; C: Pearson r = 0.9647, $p < 0.001$; E Pearson r = 0.9775, $p < 0.001$). Spread of CCS percent deviations (ΔCCS) according to most abundant adduct ions monitored with AllCCS (B), CCSbase (D) and MetCCS (F).
